# Top-Down and Middle-Down Mass Spectrometry of Antibodies

**DOI:** 10.1016/j.mcpro.2025.100989

**Published:** 2025-05-12

**Authors:** Nina A. Khristenko, Konstantin O. Nagornov, Camille Garcia, Natalia Gasilova, Megan Gant, Karen Druart, Anton N. Kozhinov, Laure Menin, Julia Chamot-Rooke, Yury O. Tsybin

**Affiliations:** 1 Spectrotech SAS, Lyon, France; 2 Spectroswiss Sarl, Lausanne, Switzerland; 3 Institut Pasteur, Université Paris Cité, and CNRS UAR2024, Paris, France; 4 Ecole Polytechnique Fédérale de Lausanne, Lausanne, Switzerland

**Keywords:** top-down mass spectrometry, middle-down mass spectrometry, monoclonal antibody, mAb, antibody-drug conjugate, ADC, proteoform, electron transfer dissociation, ETD, Orbitrap

## Abstract

Therapeutic antibodies, primarily immunoglobulin G-based monoclonal antibodies, are developed to treat cancer, autoimmune disorders, and infectious diseases. Their large size, structural complexity, and heterogeneity pose significant analytical challenges, requiring advanced characterization techniques. This review traces the 30-year evolution of top-down (TD) and middle-down (MD) mass spectrometry (MS) for antibody analysis, beginning with their initial applications and highlighting key advances and challenges throughout this period. TD MS allows for the analysis of intact antibodies, and MD MS performs analysis of the antibody subunits, even in complex biological samples. Both approaches preserve critical quality attributes such as sequence integrity, post-translational modifications (PTMs), disulfide bonds, and glycosylation patterns. Key milestones in TD and MD MS of antibodies include the use of structure-specific enzymes for subunit generation, the implementation of high-resolution mass spectrometers, and the adoption of non-ergodic ion activation methods such as electron transfer dissociation (ETD), electron capture dissociation (ECD), ultraviolet photodissociation (UVPD), and matrix-assisted laser desorption/ionization in-source decay (MALDI-ISD). The combination of complementary dissociation methods and consecutive ion activation approaches has further enhanced TD/MD MS performance. The current TD MS record of antibody sequencing with terminal product ions is about 60% sequence coverage obtained using the activated ion-ETD approach on a high-resolution MS platform. Current MD MS analyses with about 95% sequence coverage were achieved using combinations of ion activation and dissociation techniques. The review explores TD and MD MS analysis of novel mAb modalities, including antibody-drug conjugates, bispecific antibodies, endogenous antibodies from biofluids, and immunoglobulin A and M-type classes.

## Highlights

• Reviews 30 years of top-down and middle-down MS for antibody analysis.
• Highlights advances in instrumentation and ion activation techniques.
• Emphasizes improvements in sequence coverage and PTM characterization.
• Discusses TD/MD MS applications to IgG and novel antibody formats, including ADCs.
• Covers recent trends in sample preparation, data acquisition, and analysis workflows.

## Graphical Abstract

**Figure:**
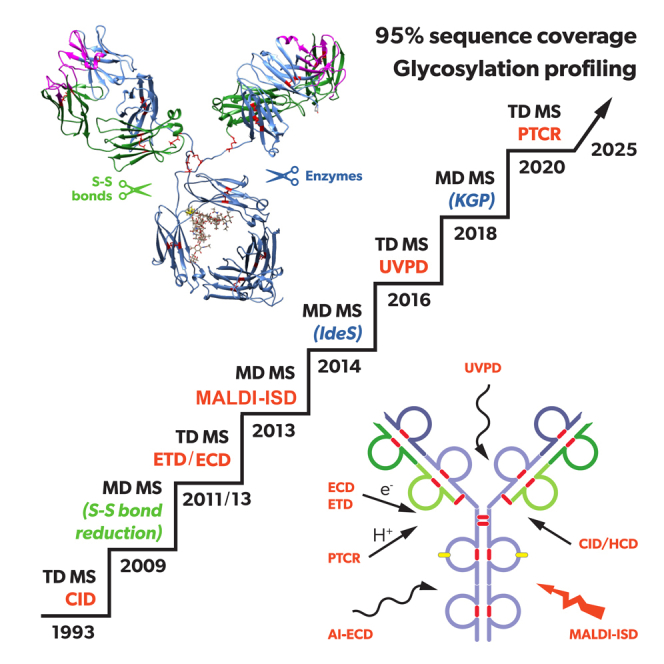

## In Brief

This review traces 3 decades of advancements in top-down and middle-down mass spectrometry (TD/MD MS) for antibody characterization. It highlights key technological and methodological developments enabling detailed analysis of intact antibodies and subunits, including sequence coverage, post-translational modifications, and structural heterogeneity. The review also explores emerging trends in instrumentation, data analysis, and applications to novel antibody formats, making TD/MD MS a powerful and evolving platform for therapeutic antibody analysis.

**Fig. 1:**
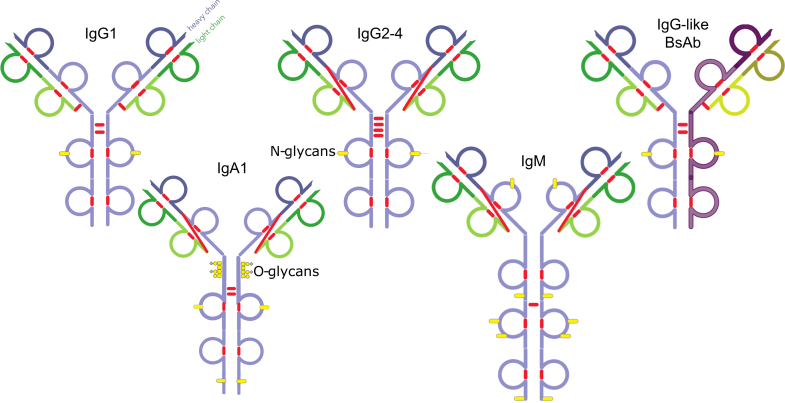
**Schematic overview of the different immunoglobulin (Ig) structures that have been studied with TD/MD MS: IgG1-4, IgA1, and IgM monomer.** These Ig structures differ in disulfide bond connectivity and glycosylation sites and type. *Red marks* represent disulfide bonds and *yellow marks* indicate N-glycosylation sites. The IgG2-4 subclasses differ in the sequence of the heavy chain’s hinge region domains.

Therapeutic antibodies (Abs), primarily immunoglobulin G (IgG)-based monoclonal antibodies (mAbs), are engineered to target specific molecules or cells to treat diseases such as cancer, autoimmune disorders, and infectious (1, 2, 3, 4). IgG-based Abs are classified into four subclasses: IgG1, IgG2, IgG3, and IgG4 (5). Their diversity arises from variations in amino acid sequences, disulfide bond connectivity, post-translational modifications (PTMs), and glycosylation patterns, all of which influence structure, stability, efficacy, and pharmacokinetics (6, 7). Beyond mAbs, antibody-drug conjugates (ADCs) (8) and engineered antibodies, including bispecific antibodies (BsAbs) (9), nanobodies, and single-chain antibodies, are expanding therapeutic possibilities (10, 11). Emerging Ab types, including IgA and IgM, are being investigated for their role in mucosal immunity and complement activation (5). This review will primarily focus on the analysis of IgG1 mAbs, while also addressing various other antibody modalities, Figure 1.

**Fig. 2:**
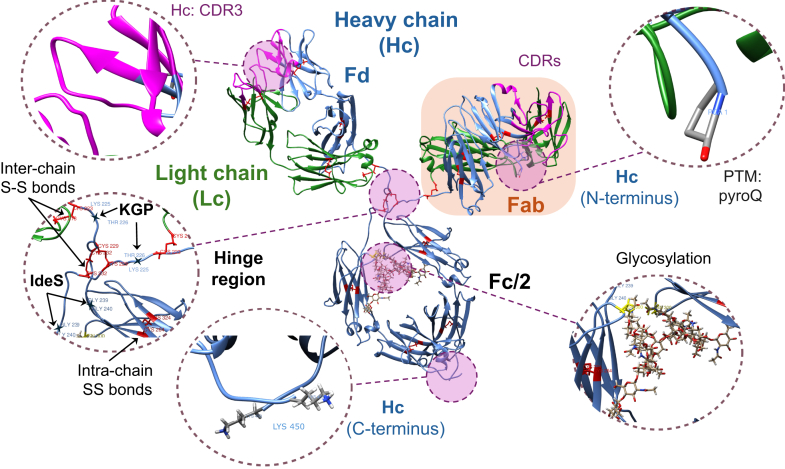
**Monoclonal antibody structural organization shown on the example of a NIST mAb.** The structural components of the NIST mAb are depicted, showing the light chain (Lc), the heavy chain (Hc), the N-terminal part of the heavy chain (Fd), and the C-terminal part of the heavy chain (Fc/2) subunits, along with the Fab region and highlighted CDRs (137). Intra- and inter-chain disulfide bonds are marked in red. The expanded views outline the following structural features: (i) CDR3 of the heavy chain, (ii) the hinge region with cleavage sites for the IdeS and KGP enzymes, (iii) the N-terminus of the heavy chain modified with a pyroglutamate, (iv) the C-terminus of the heavy chain that is prone to the loss of Lys residue, and (v) the NST glycosylation sites on asparagine residues in the Fc region (*yellow sticks*) with complex N-linked glycans.

Structural analysis of IgG-based mAbs is essential for ensuring their quality and efficacy (1). They are complex glycoproteins of about ∼150 kDa molecular weight (MW) that consist of two identical heavy (Hc) and two light chains (Lc) linked by disulfide bonds, forming a Y-shaped structure (Fig. 1). Beyond the arrangement of their chains, mAbs are defined by two distinct functional regions: the Fab (antigen-binding fragment) and the Fc (crystallizable fragment), Figure 2. The Fab region contains the hypervariable complementarity-determining regions (CDRs), which are responsible for antigen recognition, whereas the Fc region mediates immune effector functions. A short, flexible peptide segment known as the hinge region allows movement and spatial rearrangement of the Fab arms relative to the Fc region. Comprehensive structural characterization of mAbs involves primary structure analysis (amino acid sequencing), N- and O-glycosylation profiling, disulfide bond connectivity, and qualitative and quantitative assessment of post-translational modifications (PTMs). Among the most prevalent PTMs are N-terminal pyroglutamic acid formation (pyroQ), C-terminal amino acid truncation (*e.g.*, Lys residue loss), deamidation, oxidation, glycation, phosphorylation, and isomerization. Assessing these modifications is important as the most subtle change (such as deamidation, for instance) can alter the structure and function of mAbs, thereby affecting stability, activity, and immunogenicity. However, determining critical quality attributes (CQAs) of mAbs with high precision remains a significant analytical challenge (4).

Mass spectrometry (MS) has revolutionized the field of mAb characterization by offering high sensitivity, precision, and structural insights (12). Today, the MS approaches employed for mAb analysis include: intact and subunit (middle-up) mass analysis, top-down (TD) and middle-down (MD) MS, and bottom-up MS analysis (4, 7). Oftentimes, several of these approaches are used in concert to deliver the most comprehensive structural analysis. Bottom-up MS relies on proteolytic digestion(s) to generate peptides for subsequent tandem (MS/MS) analysis and is the cornerstone approach for proteomics. However, while it is effective for identifying and quantifying peptides and PTMs, bottom-up MS has several key limitations (13, 14). Some PTMs may be under-represented or missed entirely in the bottom-up MS analysis. The approach also results in a loss of connectivity information such as disulfide bond linkages, which must be inferred from separate experiments. Sample preparation can introduce artifacts such as oxidation or deamidation, while quantification at the peptide level remains indirect and is often affected by ionization biases, as well as the presence of numerous enzymatically derived modified and unmodified peptides that share the same modification site. Critically, bottom-up MS cannot resolve intact proteoforms, meaning it cannot determine how PTMs or sequence variants co-occur on the same molecule, thereby missing crucial information about the diversity of the mAb population (10, 15).

To address these limitations, TD and MD MS methods are employed (13, 16). TD MS relies on the MS/MS analysis of intact proteins without prior digestion. In turn, MD MS is based on the gas phase fragmentation of antibody subunits, primarily in the 25 to 100 kDa mass range, and offers a balance between the high-level structural insights gained from intact protein MS/MS analysis and the more detailed sequence and PTMs information typically obtained from peptide-based bottom-up MS (17, 18). Two main approaches, used either independently or in combination, are used to generate mAb subunits for MD MS: disulfide bond reduction and digestion with structure-specific enzymes. The former one, S-S bond reduction, is limited to the generation of a 25 kDa Lc and a 50 kDa Hc for IgG1 mAb. The Hc is more complex than the Lc to analyze, not only because it is twice as larger but also because it is usually N-glycosylated and carries other common PTMs such as C-terminal clipping.

The breakthrough that led to a drastic improvement in MD MS performance came with the development of structure-specific and highly efficient enzymes that offered Hc cleavage in the hinge region. Initially, the immunoglobulin G-degrading enzyme of *Streptococcus pyogenes* (IdeS) (19, 20) was used to cleave IgG below the hinge region, and the cysteine protease Gingipain K (KGP) was used to cleave IgG1 above the hinge region, Figure 2 (21). Later, other enzymes with similar or comparable specificity and improved ease of use have been added, *e.g.*, IgdE (22), SpeB (23), Bdpk (FabDELLO) (24), and IdeZ. Combined with disulfide bond reduction, these structure-specific enzymes generate ∼25 kDa mAb subunits: the Lc, the N-terminal part of the Hc (Fd or Fd’, where the prime symbol (′) indicates inclusion of the hinge region), and the C-terminal part of the Hc (Fc/2), Figure 2. The MS/MS analyses of 25 kDa subunits are simplified by the isotopically resolved subunit intact mass (middle-up) measurements, fragmentation efficiency of individually isolated charge states of precursor ions, product ion detection, and facilitated data analysis (25). Moreover, such enzymes demonstrate negligible artifact introduction upon sample preparation (26).

Since the pioneering TD MS analysis of an intact mAb in 1993 (27), MS techniques for antibody analysis have evolved significantly (17, 26). Researchers have achieved enhanced antibody structure characterization with the development of high-resolution Orbitrap Fourier transform mass spectrometry (FTMS) and advancements in time-of-flight (TOF) and FT ion cyclotron resonance (FT-ICR) MS. Introduction and application of the non-ergodic ion activation methods such as electron capture dissociation (ECD), electron transfer dissociation (ETD), matrix-assisted laser desorption/ionization in-source decay (MALDI-ISD), and ultraviolet photodissociation (UVPD) have enhanced the capabilities of TD and MD MS (28), enabling improved sequencing and detailed analysis of glycosylated regions and other challenging structural features (17, 26).

Advancements in the structural analysis of mAbs have been driven by collaborations between academic researchers and leaders in the biopharmaceutical industry. Notably, pioneering work in TD/MD of mAbs was initiated and supported by Beck’s group at Pierre Fabre Laboratories in France and Zhang’s group at Amgen in the United States. Other companies, including AbbVie, GSK, Roche Diagnostics, and Sanofi, later joined them. These biopharmaceutical industrial research groups continue to drive innovation in antibody TD and MD MS analysis by conducting their own research and fueling academic efforts with challenging samples and critical questions (4).

The ultimate goal of TD and MD MS antibody analysis, as seen today, is to achieve: (i) 100% sequence coverage, meaning cleavage of protein backbone bonds between each pair of amino acids, with detection of at least one corresponding product ion, including the distinction between isoleucine and leucine; (ii) comprehensive identification of common PTMs, such as deamidation, disulfide bond localization, N- and C-terminal modifications, oxidation, and glycosylation; (iii) accurate site-specific PTM localization within the antibody sequence; and (iv) relative quantification of proteoforms. These objectives are interconnected, as achieving complete sequence coverage is essential for accurate PTM identification and localization. However, no TD/MD MS study has reported complete sequence coverage of an antibody, even for the well-characterized IgG1 subclass. Identifying and localizing PTMs remains an ongoing challenge, particularly for other Ig classes with more complex modifications and inter- and intra-chain connectivity (Fig. 1). In biopharmaceutical studies, where the antibody sequences are known, the focus shifts to confirming CDR sequences and profiling proteoforms for major modifications.

This review offers a comprehensive overview of peer-reviewed publications describing TD and MD MS for mAbs and derived species, such as ADCs, from early studies to the latest advancements. It highlights the current advances and limitations in Ab analysis and ongoing progress. Specifically, the review emphasizes how TD/MD MS techniques constantly adapt to address the heterogeneity of Abs, including PTMs such as glycosylation. Furthermore, the review considers the application of TD/MD MS to novel antibody formats, such as ADCs, BsAbs, IgA, and IgM.

## Top-Down MS of Antibodies: Method Development

**Fig. 3:**
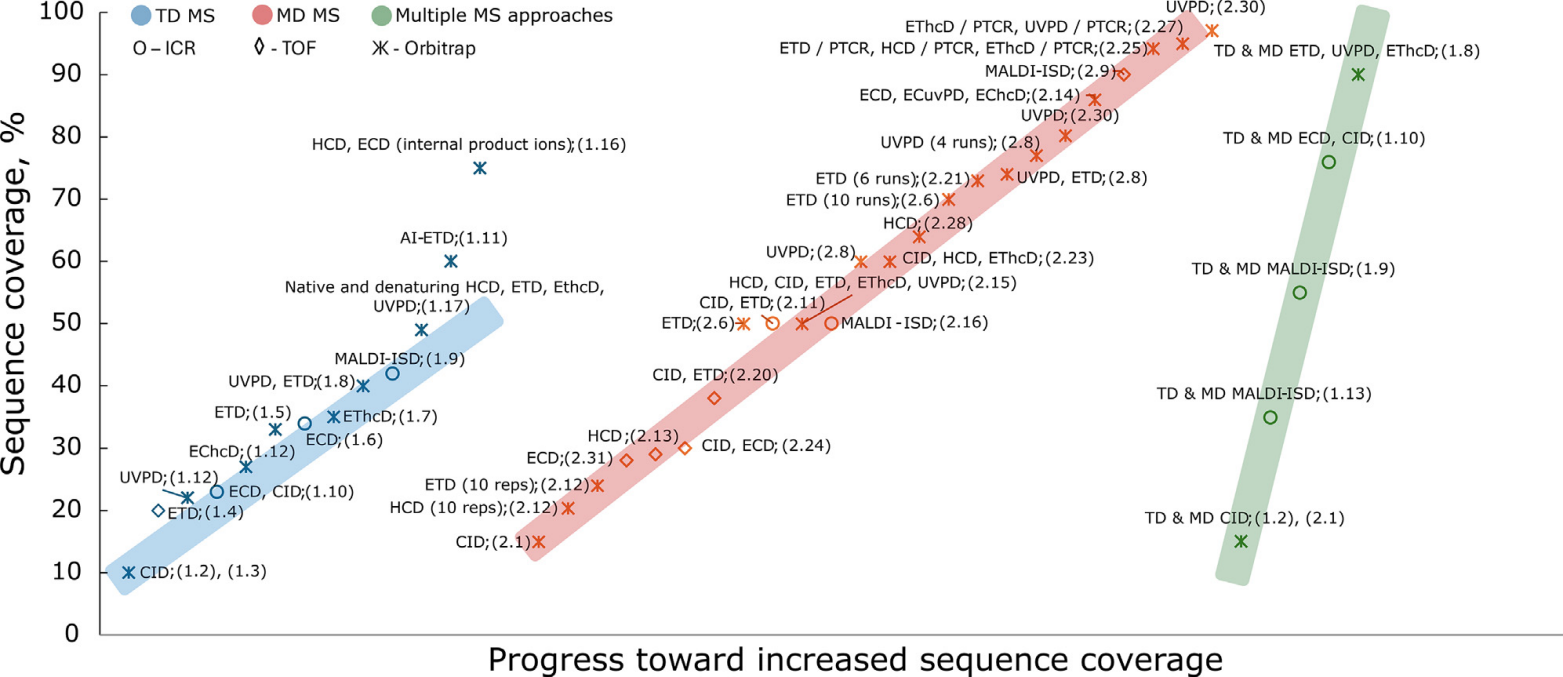
**Advances in mAbs sequence coverage annotation through diverse TD/MD MS approaches developed over the past 30 years.** Each data point corresponds to an approach with a specific MS/MS technique(s), plotted by increasing sequence coverage on the x-axis. Each point corresponds to a reference entry in Tables 1 and 2 and is labeled by the MS method and associated table row number (*e.g.*, #1.1 – row one in Table 1).

**Fig. 4:**
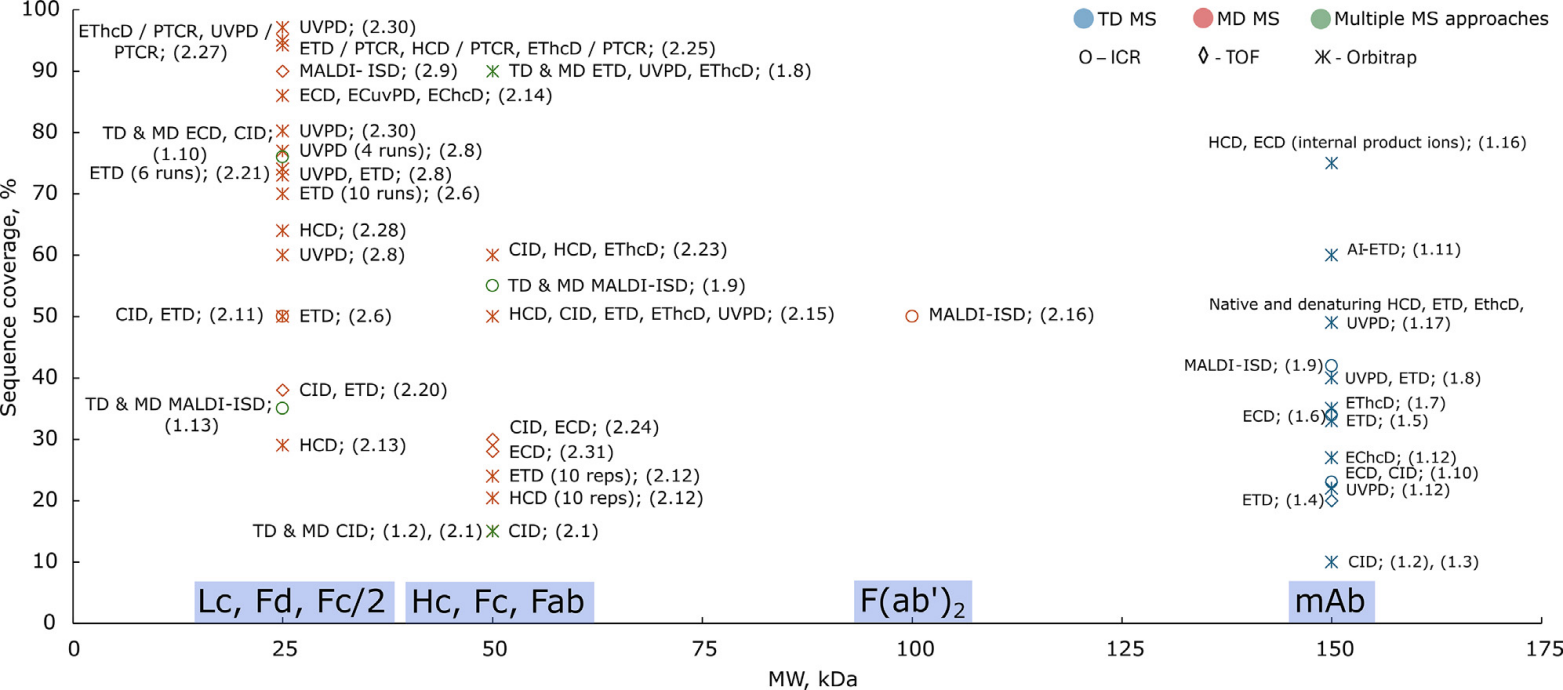
**Sequence coverage that was achieved for antibodies using TD-MS, MD-MS, and combinations of these approaches with various fragmentation techniques as a function of precursor ion molecular weight.** These molecular weights correspond to common antibody subunits generated *via* structure-specific enzymes or disulfide bond reduction. F(ab′)_2_ represents the ∼100 kDa fragment produced by IdeS digestion, containing two Lc, two Fd, and part of the hinge region. Each data point represents an experiment with a specific technique, positioned according to its resulting sequence coverage. Each point marks a specific experiment and is annotated with the method and table row number (*e.g.*, #1.1 = Table 1, row 1).

**Fig. 5:**
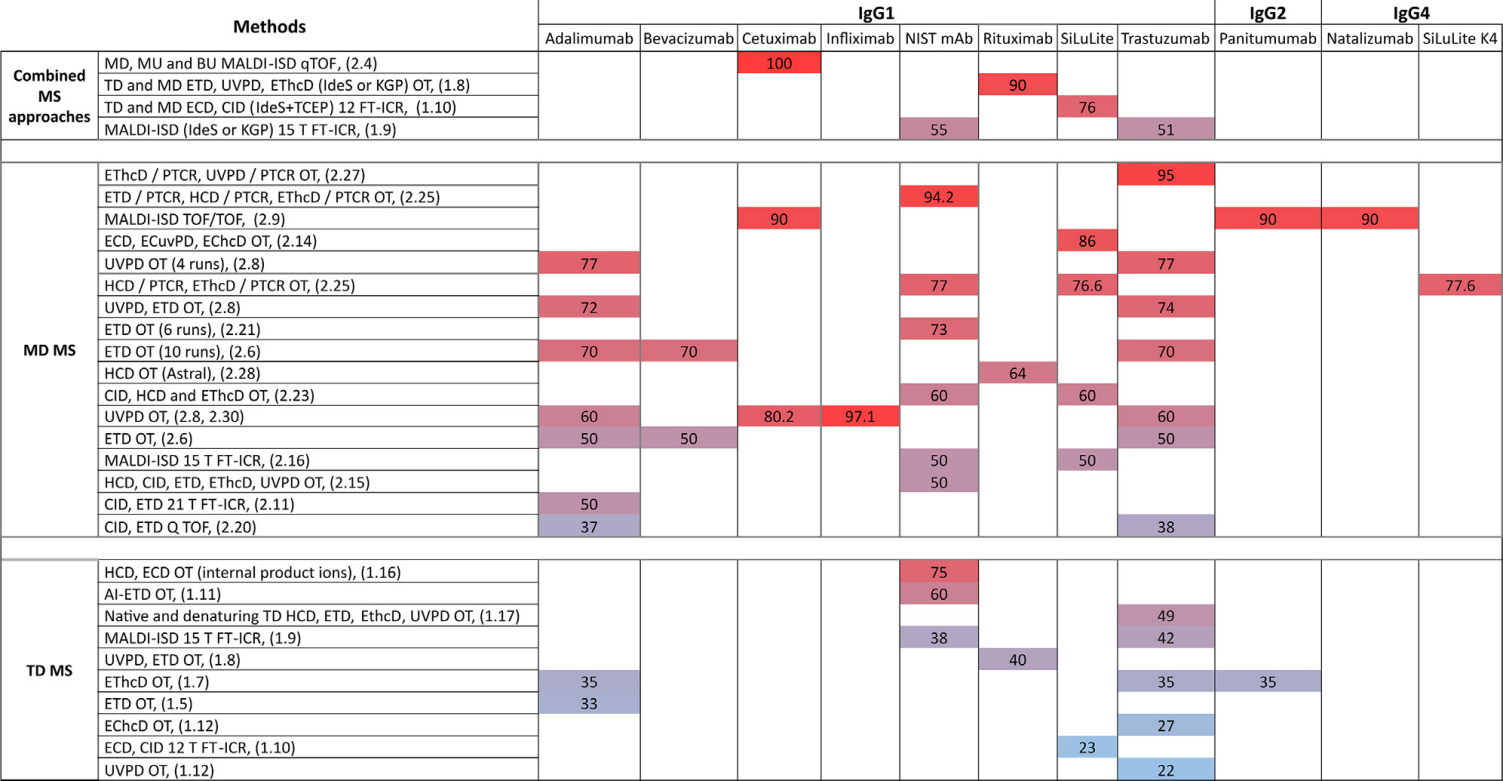
**Sequence coverage (%) obtained for mAbs over the past 30 years using TD MS, MD MS, and combinations of these MS approaches with different fragmentation techniques.** Each colored block represents a specific study, annotated with the applied MS method and the corresponding entry number from the review tables (*e.g.*, #1.1 – row one in Table 1). The percentage of sequence coverage obtained in each study is displayed within its respective block.

Table 1 and Fig. 3, Fig. 4, Fig. 5 summarize the advancements in mAbs sequence coverage achieved through diverse TD MS approaches reported over the past 30 years. In the following sections, references to entries in the tables will be indicated by the corresponding row number (*e.g.*, row #1.1 or simply #1.1).

**Table 1:** TD MS analysis of mAbs with single or multiple activation methods

#	mAb or brand name	Separation method	MS/MS method	Instrument	Sequence coverage	Ref.
1.1	Murine anti-(human q-acid glycoprotein) mAb (IgG1)	DI	CID	Triple quadrupole MS (API III MS/MS system, Sciex)	N/A	Feng and Konishi 1993 (27)
1.2	Amgen mAb (IgG1 and IgG2)	Off-line SEC - LC	isCID	LTQ Orbitrap (TFS)	<10%	Zhang and Shah 2007 (29)
1.3	Amgen mAb (IgG2)	DI and RPLC	isCID	LTQ Orbitrap (TFS)	<10%	Bondarenko et al. 2009 (30)
1.4	Human anti-Rhesus D mAb (IgG1) Murine myeloma mAb (IgG1)	RPLC	ETD	maXis UHR Q-TOF MS (Bruker Daltonics)	20%	Tsybin et al. 2011 (31)
1.5	Adalimumab (IgG1)	RPLC	ETD	LTQ Orbitrap Velos Pro (TFS)	33%	Fornelli et al. 2012 (33)
1.6	Recombinant humanized mAb (IgG1)	DI	ECD	9.4 T FT-ICR MS (NHMFL)	34%	Mao et al. 2013 (35)
1.7	Adalimumab (IgG1) Trastuzumab (IgG1) Panitumumab (IgG2)	DI and RPLC	EThcD	LTQ Orbitrap Elite (TFS)	∼35%	Fornelli et al. 2017 (37)
1.8	Rituximab (IgG1)	RPLC	UVPD, ETD	Fusion Lumos Orbitrap (TFS) – 213 nm UVPD	∼40% (3 runs)	Fornelli et al. 2018 (38)
		RPLC **TD and MD** (IdeS or KGP)	ETD, UVPD, EThcD		∼90% (6 LC-MS runs)	
1.9	NIST mAb (IgG1) Trastuzumab (IgG1)	MALDI	MALDI-ISD	15 T solariX XR FT-ICR MS equipped with a CombiSource and a ParaCell (Bruker Daltonics)	38–42%	van der Burgt et al. 2019 (40)
		MALDI **TD and MD** (IdeS or KGP)			51–55%	
1.10	SiLuLite (MSQC4) (IgG1)	DI **TD**	ECD, CID	12 T solariX FT-ICR MS (Bruker Daltonics)	23%	Jin et al. 2019 (36)
		DI **TD** and RPLC for **MD** (IdeS + endoglycosidase + TCEP)			76%	
1.11	NIST mAb (IgG1)	DI	AI-ETD	Fusion Lumos Orbitrap (TFS) modified with a Firestar T-100 Synrad 60-W CO2 continuous wave laser	60%	Lodge et al. 2020 (41)
1.12	Trastuzumab (IgG1)	nTD: DI	EChcD	Q Exactive HF/UHMR Orbitrap (TFS) modified to enable UVPD (157 nm) and ExD cell (e-MSion)	27%	Shaw et al. 2020 (43)
			UVPD		22%	
		DI **TD and MD (IdeS + KGP)**	EChcD, UVPD		CDR	
1.13	Ang-2-VEGF BsAb	MALDI **TD and MD (IdeS + DTT)**	MALDI-ISD	15 T solariX XR FT-ICR MS equipped with a CombiSource and a ParaCell (Bruker Daltonics)	∼35%	Gstöttner et al. 2020 (63)
		RPLC **BU**	HCD, ETD	Orbitrap Lumos (TFS)	81%	
1.14	Monomer IgM Pentamer (IgM)_5_J Hexameric (IgG1)_66_	DI	ECD	Q Exactive UHMR Orbitrap (TFS) with ExD cell (e-MSion)	CDRs	Greisch, den Boer, Lai, et al. 2021 (44)
1.15	anti-CD20 (IgG1, IgA1)	DI **nTD and MD** (IgdE)			CDRs	Greisch, den Boer, Beurskens, et al. 2021 (45)
1.16	NIST mAb (IgG1) ADCs (IgG1)	DI	ECD, HCD	Q Exactive plus UHMR Orbitrap (TFS) with ExD cell (e-MSion)	75% (with internal product ions)	Wei et al. 2023 (46)
1.17	Trastuzumab (IgG1)	DI **Native and denaturing TD**	HCD, ETD, EThcD, UVPD	Orbitrap Eclipse (TFS)	49%	Oates, Lieu, Srzentic, et al. 2024 (39)

A “run” refers to repeated analyses conducted under different experimental conditions, and a “replicate” refers to repeated analyses performed under identical experimental conditions (DI - direct injection, TFS - Thermo Fisher Scientific).

### Collision-Induced Dissociation (CID) TD MS

The first instance of TD MS applied to an intact mAb occurred 30 years ago (27). In this original study, Feng and Konishi attempted to gain structural information on a mAb with a triple quadrupole mass spectrometer (QQQ MS). They applied an all-ion collision-induced dissociation (CID) to fragment the intact 150 kDa species, followed by product ion detection. However, the obtained structural information was limited (#1.1 in Table 1), demonstrating the need for more advanced MS instrumentation. Significant developments in CID-based TD MS occurred 14 years later with the advent of high-resolution Orbitrap and TOF mass spectrometers, which became available in biopharmaceutical industrial laboratories.

In 2007, Zhang and Shah employed the in-source CID (isCID) fragmentation of all charge states of a mAb on a legacy LTQ Orbitrap (Thermo Fisher Scientific) to yield *b/y* product ions covering the terminal 100 to 120 residues of the Lc and Hc (29). Although an intact mAb was introduced into the mass spectrometer’s ion source, no precursor ion selection was carried out. Nevertheless, backbone cleavage events between the variable and constant domains led to the formation of *b*-type ions that retained the entire variable domain, enabling its characterization through the resulting product ions. These large *b*-ions were then submitted for MS/MS with CID in the linear ion trap (LTQ) for deep sequencing (the MS^3^ level or a pseudo-MS^3^ level, as there was no precursor ion isolation in the isCID event). The method was applied to analyze mAbs after forced oxidation with tert-butyl hydroperoxide, which successfully localized the oxidized methionine residue. In these experiments, weak backbone bonds were mostly cleaved, resulting in sequence coverage that did not exceed 10% (#1.2 in Table 1, Figs. 3 and 4). Two years later, in 2009, Zhang’s group, in collaboration with Thermo Fisher Scientific, extended this experimental approach to an IgG2 mAb, yielding similar results (#1.3 in Table 1, Figs. 3, and 4) (30).

### Electron Transfer Dissociation (ETD) TD MS

Introducing electron transfer dissociation (ETD) on high-resolution mass spectrometers was a breakthrough in TD MS analysis of intact mAbs. In 2011, the first TD MS application of ETD for the analysis of intact 150 kDa mAbs was performed using a high-resolution TOF MS (maXis Q-TOF MS, Bruker Daltonics) in a collaborative effort between Tsybin’s group and Bruker Daltonics (31). They analyzed intact IgG1 species from murine myeloma cells and human anti-Rhesus D IgG1 species, including *via* isolation of precursor ions before ion activation and dissociation. Fragmentation of several isolated charge states of intact mAb species resulted in a sequence coverage of up to 20% for both Lc and Hc (#1.4 in Table 1, Figs. 3, and 4). Fragmentation patterns revealed mAb structural influences on the MS/MS efficiency: While several sites demonstrated enhanced propensity to the backbone cleavage, the S-S bond-protected regions were rarely sequenced. An expected increase in the baseline around the abundant remaining charge-reduced precursor ions (presumably, non-covalently bound electron transfer product ions, *i.e.*, ETnoD species (32)), along with overlapping product ions, was also responsible for limited sequence coverage.

To minimize baseline elevation around intense ion signals, caused by peak interference between product and precursor ions, including undissociated charge-reduced ETnoD species, a several-fold increase in mass spectral resolution was required. This was achieved by performing TD MS with ETD on a high-resolution Orbitrap platform, LTQ Orbitrap Velos, jointly by Tsybin’s group and Thermo Fisher Scientific (#1.5 in Table 1 and Fig. 3, Fig. 4, Fig. 5) (33). Importantly, to enhance the performance of TD MS on the LTQ Orbitrap Velos, several methodological advancements were introduced or optimized, including: (i) TD MS measurements performed using an online LC-MS/MS experiment, supporting on-line sample purification and (limited) separation; (ii) reduced pressure in the C-trap, which later resulted in the development of the “protein mode” on the Orbitrap platforms; (iii) HCD-trapping used to assist in precursor ion transfer and, potentially, an early realization of the EThcD conditions; and (iv) acquisition and averaging of the unreduced data (time-domain ion signals or transients) from multiple technical replicates of the LC-MS/MS runs for the enhanced sensitivity and confidence in product ion detection. As a result, sequence coverage was almost doubled to reach 33% overall for a similar IgG1 species. Glycosylation of the Hc was not characterized, similar to the earlier ETD TOF MS measurements.

### Electron Capture Dissociation (ECD) FT-ICR TD MS

Although the added- value of ECD in the analysis of intact proteins was demonstrated as early as 1998 (34), its application to mAb analysis only emerged 15 years later and after the promising results obtained with ETD. In 2013, Marshall and co-workers at the National High Magnetic Field Laboratory (NHMFL) utilized ECD on a custom-built high-resolution 9.4 T FT-ICR MS for the direct infusion analysis of a purified 148 kDa mAb (35). Using the isolated single 51+ charge state, they achieved 25% sequence coverage (#1.6 in Table 1, Figs. 3 and 4). Results obtained in 2019 by Ying Ge and colleagues using a commercial ECD-enabled 12 T FT-ICR MS platform (SolariX XR, Bruker Daltonics) remained comparable. Ge’s team, also performing single-charge state TD MS analysis with ECD and CID, identified 172 product ions and achieved 23% sequence coverage for the SiLuLite mAb (#1.10 in Table 1 and Fig. 3, Fig. 4, Fig. 5) (36).

In the same 2013 study, Marshall and co-workers reported an increased sequence coverage of 34% when ECD was applied on several charge states (#1.6 in Table 1, Figs. 3, and 4). These advances aligned with the results obtained using Orbitrap ETD for multiple charge states regarding total sequence coverage, cleavage preferences, and backbone cleavage localization. Additionally, Marshall and co-workers activated precursor ions with infrared photons before the ECD reaction (AI-ECD); however, this did not substantially enhance performance. The authors suggested that the high charge states of product ions caused sufficient Coulombic repulsion to disrupt non-covalent precursor or product ions interactions, reducing the need for additional activation (35).

### ETD with Supplemental Ion Activation (EThcD/ETciD) TD MS

Although Tsybin and co-authors improved mAb sequence coverage using ETD by incorporating multiple charge states, varying ion-ion reaction times, and averaging several thousands of tandem mass spectra (*via* transients), the ETD-based TD MS approach still required further optimization (33). One proposed reason for the relatively limited mAb sequence coverage in ETD is the presence of non-covalent interactions between product ions (*c/z*-type), which hinder their separation and result in non-dissociative electron transfer (ETnoD). This behavior is particularly relevant for precursor ions with low charge density (*e.g*., one charge per 1000 Da), where secondary structure and disulfide bonds remain intact. As a next step, Tsybin’s group increased the additional vibration energy to the ion population following ETD activation, combining ETD and higher energy collisional dissociation (HCD), known as EThcD (37). The ETD- and EThcD-based TD MS analysis was applied to two IgG subclasses, IgG1 and IgG2, which differ in the number of interchain disulfide bonds within the antibody hinge region. The results demonstrated comparable sequence coverage of approximately 35% and similar patterns of backbone cleavage sites between the two IgG subclasses (#1.7 in Table 1 and Fig. 3, Fig. 4, Fig. 5).

Therefore, the original and follow-up results of CID- and ETD/ECD-based TD MS applied to the intact IgG1 analysis improved sequence coverage but left the glycosylation localization uncharacterized and S-S bond-protected regions largely uncovered. Additional vibrational activation, such as AI-ECD or EThcD, led to only a modest improvement in sequence coverage.

### UV Photodissociation (UVPD) and ETD TD MS

In 2018, Kelleher and colleagues set a new benchmark for IgG1 analysis, achieving approximately 40% sequence coverage of intact rituximab in TD MS experiments by combining UVPD and ETD techniques (#1.8 in Table 1 and Fig. 3, Fig. 4, Fig. 5) (38). They identified product ions with a mass accuracy of 10 ppm, as in previous studies, but enhanced their data analysis by incorporating deconvolution-free product ion validation using TDValidator (Proteinaceous). Product ion annotation was manually curated, and those that did not match the isotopic fitter, *e.g.*, detected with fewer than three correctly matched isotopologues for a large product ion, were not considered.

In 2024, an UVPD-related report from the Fornelli group compared native and denaturing TD MS analysis of trastuzumab (39). They benchmarked four fragmentation techniques: HCD, ETD, EThcD, and 213 nm UVPD. In their study, native TD MS achieved slightly higher sequence coverage than denaturing TD MS when using HCD and ETD, yielded comparable results with EThcD, and showed marginally lower coverage with UVPD (#1.17 in Table 1 and Fig. 3, Fig. 4, Fig. 5). Although more precursor ions were selected for native TD MS (due to the same AGC value used in both native and denaturing TD MS, with lower precursor charge states in the native analysis), the findings suggest the added value of native TD MS for primary structure analysis applications.

### MALDI In-Source Decay (ISD) TD MS

In 2019, MALDI-ISD was applied in the TD MS analysis of two antibodies: trastuzumab and the NIST mAb standard. The TD MS antibody analysis was conducted using a high-resolution 15 T FT-ICR MS (SolariX XR, Bruker Daltonics). The data analysis benefited from the enhanced resolution of absorption mode FT mass spectra, a technique adopted by Nicolardi's group and enabled by transient post-processing using the AutoVectis software package (40). As a result, TD MS analysis with a single activation method achieved an increased sequence coverage for trastuzumab and the NIST mAb standard at 38% and 42%, respectively (#1.9 in Table 1 and Fig. 3, Fig. 4, Fig. 5).

### Activated Ion Electron Transfer Dissociation (AI-ETD) TD MS

In 2020, the range of ETD-based TD methods applied to the intact mAbs analysis was expanded with the introduction of activated ion ETD (AI-ETD), developed by the Coon’s group (41). In AI-ETD, precursor ions are irradiated with infrared photons before or during the ETD event, which helps break non-covalent interactions between product ions, thus enhances fragmentation efficiency. This concept was originally demonstrated in AI-ECD experiments for peptides and smaller proteins (42). However, in comparison with the limited performance improvement with AI-ECD of mAbs in FT-ICR MS, the Coon’s group achieved a 60% sequence coverage of intact mAbs using TD MS analysis with AI-ETD on a hybrid Orbitrap platform (Orbitrap Fusion Lumos, Thermo Fisher Scientific), row #1.11 in Table 1 and Fig. 3, Fig. 4, Fig. 5. Notably, varying the reaction conditions (*e.g.*, from 18 W, 20 ms to 24 W, 180 ms) produced different types of product ions, offering deeper insights into disulfide-bonded regions (41).

### Electron Capture Dissociation (ECD) Orbitrap TD MS

During the same year, 2020, Voinov, Shaw, and colleagues reported on a method for cleaving the disulfide bonds that link the Lc and Hc subunits of trastuzumab using ECD with a supplemental activation *via* higher energy collisions (EChcD) or *via* UVPD at 157 nm, or ECuvPD (43). Building on this concept, they demonstrated that isolating the intact antibody or its Fab subunit and performing on-the-flight disulfide bond reduction enabled direct determination of chain pairing, *i.e.* revealing which Lc is connected to which Hc (precise localization of the involved cysteines is not needed). This approach can be particularly valuable for analyzing complex antibody mixtures containing diverse light and heavy chain pairings. Their setup featured a modified Q Exactive HF/ultra-high mass-range (UHMR) Orbitrap mass spectrometer (Thermo Fisher Scientific) integrated with an electromagnetostatic E x D cell (e-MSion Inc., Corvallis, Oregon). These analyses yielded reliable identification of 27% and 22% of the antibody sequences with an improved mass tolerance of 5 ppm using EChcD and ECuvPD activation methods, respectively (#1.12 in Table 1 and Fig. 3, Fig. 4, Fig. 5). Notably, this study highlighted the advantages of performing native antibody MS analysis and selecting low-charge-state precursor ions, which helped to reduce spectral congestion and minimize interferences for product ions. Chronologically, the chain pairing TD MS application followed the original MD MS approach developed by Tsybin and co-workers in 2018, which utilized internal product ions for mAb chain pairing (#2.12 in Table 2, Figs. 3, and 4).

**Table 2:** MD MS analyses of mAbs with single or multiple activation methods

#	mAb or brand name	Sample preparation	Separation method	MS/MS method	Instrument	Sequence coverage	Ref.
2.1	Amgen mAb (IgG2)	DTT	RPLC	CID	LTQ Orbitrap (TFS)	∼15%	Bondarenko et al. 2009 (30)
2.2	Amgen mAb (IgG)	TCEP	RPLC	isCID	LCT Premier TOF MS (Waters)	N/A	Ren et al. 2009 (47)
2.3	Nanobody (IgG heavy chain V_H_)	DNA MALDI matrix or TCEP	MALDI	MALDI-ISD	ultrafleXtreme MALDI-TOF/TOF MS (Bruker Daltonics)	100% (V_H_)	Resemann et al. 2010 (56)
2.4	Cetuximab (IgG1)	**MD, MU, and BU** (IdeS + TCEP)	DI or RPLC	MALDI-ISD BU: MALDI-ISD and ESI CID	MU: maXis 4G high resolution Q-TOF MD: rapifleX TOF/TOF bottom-up: LC-ESI- maXis impact high resolution Q-TOF and MALDI ultra-fleXtreme TOF/TOF (all Bruker Daltonics)	100%	Ayoub et al. 2013 (58)
2.5	Roche Diagnostics mAbs (IgG1)	Electrochemical cell	DI	CID	15 T solariX FT-ICR MS (Bruker Daltonics)	N/A	Nicolardi et al. 2014 (48)
2.6	Adalimumab (IgG1) Bevacizumab (IgG1) Trastuzumab (IgG1)	IdeS + TCEP	RPLC	ETD	LTQ Orbitrap Elite (TFS)	∼50% (1 run) ∼70% (10 runs)	Fornelli et al. 2014 (52)
2.7	Adalimumab (IgG1) Alemtuzumab (IgG1)	**MD and BU** (Papain + TCEP)	Chromato-focusing and RPLC	ETD, HCD	12 T Solarix FT-ICR MS (Bruker Daltonics) in-house ICR cell and 15 T Solarix FT-ICR MS (Bruker Daltonics) LTQ Orbitrap Velos and Q Exactive (TFS)	100% (Fab)	Dekker et al. 2014 (64)
2.8	Trastuzumab (IgG1) Adalimumab (IgG1)	IdeS + TCEP	RPLC	UVPD	LTQ Orbitrap Elite (TFS) outfitted with a 193 nm ArF excimer laser (Coherent ExciStar XS)	∼60% (1 run) ∼77% (4 runs)	Cotham and Brodbelt 2016 (67)
				UVPD + ETD		72–74%	
2.9	Cetuximab (IgG1) Panitumumab (IgG2) Natalizumab (IgG4)	EndoS + IdeS + TCEP/DTT + for Cetuximab PNGase F	MALDI and RPLC	MALDI-ISD	ultrafleXtreme MALDI-TOF/TOF (Bruker Daltonics)	∼90%	Resemann et al. 2016 (59)
2.10	Cetuximab (IgG1)	IdeS	CE	MALDI-ISD	Autoflex II MALDI-TOF (Bruker Daltonics)	9% (Fc/2)	Biacchi et al. 2017 (61)
2.11	Adalimumab (IgG1)	TCEP or IdeS + TCEP	RPLC	CID, ETD	21 T LTQ FT-ICR MS (NHMFL)	∼50%	He et al. 2017 (65)
2.12	Trastuzumab (IgG1 Fab)	KGP	RPLC	ETD	LTQ Orbitrap Elite (TFS)	∼24% (10 reps)	Srzentić et al. 2018 (50)
				HCD	Q Exactive HF Orbitrap (TFS)	20.4% (10 reps)	
2.13	IgG1	IdeS or IdeS + TCEP	CZE	HCD	Q Exactive Plus Orbitrap (TFS)	29%	Belov et al. 2018 (51)
2.14	SiLuLite mAb(MSQC4) (IgG1)	IdeS + DTT	RPLC	ECD, ECuvPD, EChcD	Q Exactive HF Orbitrap (TFS) modified to enable UVPD in the HCD cell and an ExD cell (e-MSion)	86%	Shaw et al. 2018 (69)
2.15	NIST mAb (IgG1)	TCEP	FAIMS	HCD, CID, ETD, EThcD, UVPD	Orbitrap Eclipse with FAIMS Pro (TFS)	∼50%	Melani et al. 2019 (71)
2.16	SiLuLite mAb (IgG1) NIST mAb (IgG1)	IdeS	MALDI	MALDI-ISD	15 T solariX XR FT-ICR MS with a CombiSource and a ParaCell (Bruker Daltonics)	∼50%	Nicolardi et al. 2020 (62)
2.17	ADC from AbbVie	IdeZ + TCEP	RPLC	UVPD-PTCR	Orbitrap Fusion Lumos (TFS) with a 193 nm excimer laser (Coherent Excistar XS)	74% (Lc + 1 payload)	Sanders et al. 2020 (72)
2.18	SiLuLite (IgG1) NIST mAb (IgG1) Trastuzumab (IgG1)	TCEP	RPLC	CID/PTCR + ETD/PTCR	21 T LTQ FT-ICR MS (NHMFL)	85% (Lc)	Srzentić et al. 2020 (14)
2.19	IgG1 - 4	IdeS	DI	nTD: ECD	Q Exactive UHMR Orbitrap (TFS) with ExD cell (e-MSion)	CDR3	den Boer et al. 2020 (53)
2.20	Trastuzumab (IgG1) Adalimumab (IgG1)	IdeS + TCEP	SEC- native RPLC- denaturing	CID, ETD	maXis II ETD UHR Q TOF MS (Bruker Daltonics)	38%	Zhu, Li, and Zhang 2021 (66)
2.21	NIST mAb (IgG1)	IdeS + DTT	RPLC	ETD	Orbitrap Fusion Lumos (TFS)	73% (6 runs)	Cejkov et al. 2021 (54)
2.22	NIST mAb (IgG1)	DTT	CE(SDS)-CZE	HCD, ETD, UVPD	Orbitrap Fusion Lumos (TFS)	64% (Lc)	Römer et al. 2021 (73)
2.23	SiLuLite (IgG1) NIST mAb (IgG1)	DTT	RPLC	CID, HCD, EThcD	Orbitrap Eclipse (TFS)	60%	Dhenin, Dupré, et al. 2023 (74)
2.24	GSKmAb biotinylated, goat-derived antihuman (IgG)	TCEP	RPLC	CID, ECD	ZenoTOF 7600 (Sciex)	30%	Kellie et al. 2023 (75)
2.25	NIST mAb (IgG1) SILuLite (MSQC4) (IgG1) SiLuLite K4 (MSQC14) (IgG4)	IdeS + DTT/TCEP	RPLC	ETD/PTCR, HCD/PTCR, EThcD/PTCR	Orbitrap Eclipse (TFS) with ETD/PTCR, high mass range	94.2% (3 runs)	Oates, Lieu, Kline, et al. 2024 (76)
2.26	SILu SigmaMab ADC (MSQC8) (IgG)	IdeS + DTT/TCEP	RPLC	ETD/PTCR, HCD/PTCR, EThcD/PTCR UVPD/PTCR	Orbitrap Ascend (TFS) with ETD, UVPD, PTCR	>79.5% (Fd with 1 or 2 payload(s))	Lieu et al. 2024 (77)
2.27	Trastuzumab (IgG1)	IdeS + DTT	SEC- native RPLC- denaturing	EThcD/PTCR, UVPD/PTCR	Orbitrap Ascend (TFS)	95% (10 runs)	Huang et al. 2024 (102)
2.28	Trastuzumab (IgG1) Rituximab (IgG1) NISTmAb (IgG1) SigmaMAb ADC Mimic (IgG1)	**Intact mass (native, denaturing), MD, BU** IdeS + TCEP	SEC- native RPLC- denaturing	HCD	Orbitrap Astral (TFS)	MD - 64%	Srzentić et al. 2024 (103)
2.29	Multiple myeloma mAb (IgG (Lc))	TCEP	RPLC	ECD	Q Exactive HF BioPharma (TFS) with an Omnitrap (Fasmatech) and FTMS Booster X2 (Spectroswiss)	93% (Lc)	Garcia et al. 2024 (121)
				EThcD	Orbitrap Eclipse (TFS)		
2.30	Infliximab (IgG1)	IdeS + TCEP	RPLC	UVPD	Orbitrap Fusion Lumos (TFS) with 193-nm ArF excimer laser (Gam laser)	97.1%	Liu et al. 2025 (68)
	Cetuximab (IgG1)					80.2%	
2.31	Intact mAb Mass Check Standard	TCEP	RPLC	ECD	ZenoTOF 7600 (Sciex)	28%	Searfoss et al. 2025 (55)

Definitions and abbreviations are as in Table 1.

In 2021, the Heck group extended the application of TD MS to the analysis of sub-million Da IgM- and IgG-based oligomeric immunoglobulins, row #1.14 in Table 1 (44). A Q Exactive UHMR Orbitrap (Thermo Fisher Scientific) equipped with the E x D cell was employed to analyze pentameric IgM and hexameric IgG1. Interestingly, the ECD mass spectra acquired for both the multimeric and monomeric (Fig. 1) forms showed only a 20% difference, suggesting that the assembly of these complexes has a low impact on product ion formation.

In a subsequent study, Heck and colleagues compared native TD MS of IgG1 and IgA1 using ECD (#1.15 in Table 1) (45). Unlike IgG1, IgA1 exhibits significantly higher glycosylation and glycan complexity (Fig. 1), leading to charge state-unresolved ion signals which complicate intact mass measurement. Despite this complexity, the researchers obtained amino acid sequence ladders, limited to *c*-ions, that covered the CDR3s and other regions. Notably, there was minimal variation between the native TD ECD mass spectra of the heterogeneously N- and O-glycosylated IgA1, the N-glycosylated IgG1, and their Fab subunits. These findings suggested that key regions like CDR3s can be comprehensively characterized in IgGs, regardless of their class or subclass, even when dealing with highly glycosylated forms.

More recently, Loo and colleagues achieved up to 75% sequence coverage of the NIST mAb by including internal product ion annotations in addition to conventional terminal fragment analysis, #1.16 in Table 1 and Fig. 3, Fig. 4, Fig. 5 (46). They employed a Q Exactive Plus Orbitrap outfitted with the UHMR and E x D cell capabilities, using both HCD and ECD to generate *b, c, y,* and *z*-type product ions in native TD MS analysis. The study underscores the valuable insights potentially gained from internal product ion annotations within an intact mAb, such as enhanced sequence coverage, identification of intra-chain and inter-chain disulfide linkages, and localization of N-glycosylation sites. To ensure high confidence in annotations, they applied stringent criteria: a 3-ppm mass tolerance and a minimum product ion length of five amino acids, along with manual validation.

## Middle-Down MS of Antibodies: Method Development

MS/MS analysis of antibody subunits, known as MD MS, retains the key advantages of TD MS, such as minimal sample preparation and reduced risk of unwanted modifications, while offering more accurate subunit mass measurements and improved sequence coverage, owing to the absence of intra- and inter-chain disulfide bonds and the smaller size of the protein subunits (30). Table 2 and Figure 4 illustrate the increase in antibody sequence coverage achieved with decreasing subunit MW from the inception of the method to the current state-of-the-art. Each data point represents a specific experiment, showing how breaking down antibodies into several smaller subunits can enhance sequence coverage across different MS approaches and fragmentation techniques.

### Collision-Induced Dissociation (CID) MD MS

In 2009, recombinant IgG1 and IgG2 antibodies were analyzed for the first time using CID-based MD MS, in conjunction with TD MS (30). In the MD MS experiments’ sample preparation, intact mAbs were separated into Lc and Hc in-solution by reducing the inter-chain disulfide bonds. These subunits were then subjected to MS/MS analysis, with CID in the LTQ and product ion detection in the Orbitrap mass analyzer. The MD MS analysis resulted in a higher number of product ions and better structural resolution of antibodies compared to the TD MS analysis; however, sequence coverage still did not exceed 15% (#2.1 in Table 2, Figs. 3, and 4).

In a parallel study, the isCID MS/MS analysis performed on separated Lc and Hc subunits using a TOF MS demonstrated comparable performance and limitations (#2.2 in Table 2), enabling the rapid sequencing of the first seven N-terminal residues and the characterization of pyroglutamic acid formation, Figure 2 (47). In addition to the low resolution of the employed TOF MS platform, among the reasons of this relatively low sequence coverage for both instruments were high mass and glycosylation heterogeneity of subunits (50 kDa for Hc) and CID cleavage preferences.

Five years later, a high-resolution FT-ICR MS instrument was also employed for CID-based MD MS analysis of the reduced IgG1 antibody. Notably, instead of the in-solution disulfide bond reduction, Nicolardi and colleagues coupled an electrochemical cell to a 15 T FT-ICR MS to perform online MD MS analysis (48). The Lc and Hc subunits of intact IgG1 mAb were separated into individual free Hc and Lc, as well as combinations of these (Lc + Hc and Lc+2Hc), using electrochemical-assisted reduction of interchain disulfide bonds. The isolated Lc precursor ions were then subjected to MS/MS analysis with CID, which confirmed that two intrachain disulfide bonds remained intact, including one located in the variable region between Cys28 and Cys88 (#2.5 in Table 2).

### Higher-Energy Collision-Induced Dissociation (HCD) MD MS

Higher-energy collision-induced dissociation (HCD) was implemented on the Orbitrap platforms already in 2007 and became widely utilized instead of CID for peptide structural analysis (49). However, as HCD MS/MS alone provided limited sequence coverage for large proteins, including intact mAbs, it has been employed as a complementary ion activation method alongside electron-based MS/MS techniques in TD/MD MS of mAbs. Like the HCD-based TD MS, the HCD-based MD MS approach generally yields fewer product ions compared to electron-based techniques, as exemplified below, necessitating the complementary use of these methods to achieve a more comprehensive analysis.

In one HCD-based MD MS study, the Tsybin and Menin groups analyzed the Fab subunits of trastuzumab by complementing the ETD-based measurements performed on the LTQ Orbitrap Elite with HCD on a Q Exactive HF mass spectrometer (50). The Lc and Fd subunits were generated by specific cleavage above the hinge region using the KGP enzyme. Data from 10 LC-MS/MS runs were processed using spectral averaging, comparing two types of FTMS data: enhanced FT (eFT, reduced profile mode) and absorption mode FT (aFT, full profile mode). Spectral averaging from the aFT mode assigned nearly twice as many *b*- and *y*-type product ions compared to eFT. Sequence coverage using HCD for the Fd subunit reached 10.7% with eFT and 20.4% with aFT. Overall, HCD provided sequence coverage exceeding 20% for the whole Fab subunit (#2.12 in Table 2, Figs. 3, and 4).

In another study, the MD MS analysis with the HCD approach enabled the characterization of the deamidated proteoforms and glycoforms of the Fc/2 domain, as well as the confirmation of specific PTM sites (51). To achieve these results, Ivanov and colleagues employed a high-resolution capillary zone electrophoresis (CZE)-MS setup to conduct both intact mass analysis (without dissociation) and MD MS analysis of antibody subunits. Intact antibody MS analysis under both native and denaturing conditions revealed different mAb populations, including doubly glycosylated, singly glycosylated, and non-glycosylated structures, along with the detection of dimers, dissociated Lc, and even Lc dimers subunit (#2.13 in Table 2, Figs. 3, and 4).

### Electron-Based Dissociation MD MS

In 2014, the IdeS enzyme was employed in the original implementation of ETD-based MD MS analysis by Tsybin’s group to cleave IgG1 below the hinge region (50, 52). As a result, sequence coverage of IgG1 increased to 70% (#2.6 in Table 2 and Fig. 3, Fig. 4, Fig. 5), and PTMs, *e.g.*, methionine oxidation, were more amenable to localization. In their follow up work on ETD-based MD MS, the same group used the KGP enzyme to cleave IgG1 above the hinge region (#2.12 in Table 2, Figs. 3, and 4). The ETD-based MD MS applied to the KGP-derived Fab subunit of trastuzumab allowed to identify chain pairing based on branched (internal) product ions, *i.e.* ions containing portions of both the heavy and light chains connected *via* a disulfide bond (50). Both MD MS studies confirmed the advantages of processing unreduced MS data, achieved through the analysis of either transients or aFT (full profile) mass spectra, which had previously proven successful in TD MS.

Heck and colleagues conducted a pure ECD-based native MD MS analysis of F(ab’)_2_ parts from four IgG subclasses cleaved using IdeS: IgG1, IgG2, IgG3, and IgG4 (53). They successfully applied a Q Exactive UHMR Orbitrap (Thermo Fisher Scientific) equipped with the E x D cell (e-MSion Inc.) as described earlier for TD MS. The generated *c-*ions produced straightforward-to-read sequence ladders, facilitating the *de novo* sequencing of IgG CDR3s (#2.19 in Table 2).

In the MD MS study listed as #2.21 in Table 2, the mAb sequence coverage was increased to 73% for entire NIST mAb (with 75%, 78%, and 64% for Fc/2, Lc, and Fd subunits, respectively) by applying a design of experiments (DoE) approach to optimize ETD parameters such as precursor ion isolation window, ETD reaction time, number of ETD reagent molecules, and number of precursor ions (*via* automatic gain control, AGC, optimization) (54). Additionally, the DoE models were developed to fine-tune ETD settings for generating product ions across different mass ranges: low (<5 kDa), medium (5–10 kDa), and high (>10 kDa). The ETD reaction time was identified as the primary factor influencing product ion size in these models. This observation aligns with TD MS-based ETD analysis of mAbs conducted by Fornelli *et al.*, which demonstrated that ETD reaction time significantly impacts the diversity of product ions (37). The DoE models confirmed that shorter ETD reaction times generally produced larger product ions. In contrast, longer reaction times resulted in a greater abundance of smaller ions in the ETD MS/MS spectra.

As an alternative for electron-based techniques coupled to Orbitrap platforms, Garcia and colleagues employed the ZenoTOF system (Sciex) using electron-activated dissociation (EAD, a commercial form of ECD) to analyze a well-characterized antibody (Waters Intact mAb Mass Check) (55). Intact mass analysis revealed five glycoforms. Subsequent MD MS analysis of the 25 and 50 kDa subunits—generated *via* disulfide bond reduction—achieved 28% overall sequence coverage (21% for the heavy chain and 42% for the light chain) (#2.31 in Table 2, Figs. 3, and 4).

### MALDI In-Source Decay (ISD) MD MS

In 2010, MALDI-based in-source decay (ISD) approach was added to the arsenal of MS/MS methods employed for MD MS of mAbs. Suckau and colleagues from Bruker Daltonics analyzed Camelidae IgG heavy chain antibodies, which lack light chains and contain a single variable domain (*V*_*H*_*H* or nanobody, ∼14 kDa) (56). The analysis was conducted using an ultrafleXtreme MALDI-TOF/TOF mass spectrometer (Bruker Daltonics) with 1,5-diaminonaphthalene (DAN) and super 2,5-dihydroxybenzoic acid (sDHB) matrices. DAN helps to reduce disulfide bonds, enhancing N-terminal (*c-*type ions) and C-terminal (*z*-type ions) fragmentation and simplifying sequence analysis. In contrast, sDHB with TCEP-reduced samples promoted stronger *c-, z-, a-,* and *y-*type ion formation. The complementary use of these matrices enabled targeted analysis of both termini, generating long sequence tags with uniform fragmentation. The singly charged nature of the resulting product ions further facilitated *de novo* sequencing by reducing spectral complexity and improving ion assignment. To extend sequence tags toward the protein’s central part, a second level of sequence analysis, pseudo-MS^3^ or deep sequencing, was performed on the same MALDI-TOF/TOF instrument (57). This pseudo-MS^3^ approach further improved sequence coverage and terminal fragment identification. As a result, the entire 14 kDa nanobody sequence was correctly identified with 100% coverage, except for Leu/Ile ambiguities, which were resolved by bottom-up MS analysis (#2.3 in Table 2).

In 2013, the Suckau group at Bruker Daltonics collaborated with Beck’s group to integrate MD MS with middle-up and bottom-up MS strategies for a comprehensive analysis of the primary structure and glycosylation profiling of cetuximab (58). This study utilized four high-resolution TOF MS instruments (Bruker Daltonics), including maXis 4G LC-ESI-Q-TOF for middle-up MS, rapifleX MALDI-ISD TOF/TOF for MD MS, as well as LC-ESI-maXis impact high resolution Q-TOF and MALDI ultrafleXtreme TOF/TOF for bottom-up MS analysis. The LC-MS-based middle-up analysis enabled MW determination at the subunit level. The MALDI-ISD-based MD MS approach investigated glycosylation variants and the amino acid sequence, identifying two errors in the then-current cetuximab sequence: a missing Cys_214_ and an incorrectly proposed Glu_213_ instead of Ala_213_. Further peptide mapping experiments using bottom-up LC-MS/MS with CID on tryptic and GluC digests achieved 100% sequence coverage for both Lc and Hc (#2.4 in Table 2 and Fig. 5), enabling the localization of PTMs and sequence variants.

In 2016, these groups achieved an almost complete sequence validation of IgGs demonstrated using the combination of accurate mass measurements of 25 kDa IgG subunits with a ESI-based Q-TOF MS (middle-up MS approach) and MALDI ISD TOF MS/MS approach applied to the analysis of 25 kDa subunits of IgG1 (cetuximab, a chimeric IgG produced in Sp2/0 cells), IgG2 (panitumumab, a human IgG produced in CHO cells), and IgG4 (natalizumab, a humanized IgG produced in NS0 cells) (59). The antibody subunits were produced from the antibodies deglycosylated using EndoS by digestion with IdeS and disulfide bond reduction by TCEP or DTT. An additional deglycosylation of cetuximab was performed by PNGase F enzyme. The authors outlined that the MALDI-ISD TOF MS approach resulted in a preferential sequencing of the eighth-10th residues from each terminus and exhibited a prohibited cleavage of the N-C_α_ bond of proline residues (#2.9 in Table 2 and Fig. 3, Fig. 4, Fig. 5) (59). The latter is also a fundamental characteristic of ETD/ECD approaches (60). The following year, François’ group, in collaboration with the same group of Beck from Pierre Fabre Laboratory, analyzed the Fc/2 subunit of cetuximab by coupling off-line CZE and MALDI ISD TOF MS (61). An overall sequence coverage of 9% for Fc/2 was obtained, and the detected mass shift in the *y*-ions series confirmed the CZE separation of Fc/2 dimers with and without C-terminal Lys truncation (#2.10 in Table 2).

In 2019, Nicolardi and co-workers complemented their TD MS experiments with the MD MS analysis of trastuzumab and NIST antibodies on a high-resolution 15 T FT-ICR MS instrument (40). Both IdeS and KGP enzymes were employed to generate various antibody subunits. The experiments were performed using an enhanced resolution of 660,000 at *m/z* 400 on a 15 T FT-ICR MS (SolariX XR, Bruker Daltonics). As a result, overall sequence coverage increased from 42% with TD MS alone to 55% through the integration of TD MS and MD MS data (#1.9 in Table 1 and Fig. 3, Fig. 4, Fig. 5). The confidence in product ion identification was supported by using mass tolerances of 10, 20, and 40 ppm for *m/z* ranges of 1012 − 5000, 1012 − 7000, and 2024 − 30,000, respectively. This study also provided insights into N-glycosylation of the Fc region in trastuzumab and NIST antibodies, with the most abundant glycoforms of the Fc regions detected as doubly charged ions and identified at isotopic level resolution.

One year later, Nicolardi and co-workers analyzed another pair of antibodies, SiLuLite and NIST, using MALDI-ISD on the same high-resolution 15 T FT-ICR MS (SolariX XR, Bruker Daltonics) both in positive and negative modes (62). While positive mode required presence of basic residues for extensive sequence readout, negative mode was particularly effective for antibodies with acidic residues in N- and C-termini sequences, extending the sequenced regions from both termini. This complementary approach enabled to achieve an overall sequence coverage of ∼50% (#2.16 in Table 2 and Fig. 3, Fig. 4, Fig. 5).

In 2020, the Nicolardi group conducted an in-depth investigation of the primary structure and glycation of a BsAb, a recombinant protein with distinct Lc and Hc designed to bind two different epitopes or antigens (63). They employed TD and MD MS approaches using MALDI-ISD on a 15 T FT-ICR MS (SolariX XR, Bruker Daltonics) and bottom-up MS using ESI-based LC-MS Lumos Orbitrap with HCD. In contrast to classical single-specificity mAb MD MS analysis, six different antibody subunits were obtained after IdeS digestion and chemical reduction of disulfide bonds. This approach successfully tracked glycation changes during a 168-h experiment and localized glycation hotspots by combining results from TD MS, MD MS, and bottom-up MS analyses. This work revealed 34% sequence coverage with TD/MD MS and 81% with bottom-up MS for the Ang-2-VEGF BsAb (#1.13 in Table 1 and Figs. 3, and 4).

## Combined MS/MS and TD/MD MS Techniques

In the first section, we describe the early efforts to combine methods like CID, HCD, ETD, and UVPD for enhanced sequence coverage in mAbs and mAbs subunits. The next section focuses on the synergy between TD and MD MS, emphasizing complete structural insights through complementary methods. Finally, we highlight technological innovations and optimization strategies that have pushed the limits of TD and MD MS.

### Combining Fragmentation Techniques

The combination of fragmentation techniques was first exemplified in 2014 by Paša-Tolić and colleagues who improved sequence coverage of adalimumab’s Fab subunit using HCD and ETD in LC-based MD MS analyses (64). For the Fd’ subunit, expectedly, HCD resulted in 23% coverage, and ETD achieved 46%, leading to an overall combined Fd’ sequence coverage of 53%. For the Lc, HCD alone provided a higher sequence coverage (46%) compared to ETD (28%), yielding a combined sequence coverage of 64%. The integration of MD and bottom-up MS approaches enabled complete Fab subunit sequence characterization for adalimumab and alemtuzumab (64). First, authors obtained antibody subunits *via* digestion with papain and successfully separated the Fabs of the two antibodies by utilizing a 2D LC approach, specifically chromate-focusing followed by RP separation, and coupling it with high-resolution FT-ICR and Orbitrap mass spectrometers. Second, through database searches using the Fab subunit sequences of adalimumab and alemtuzumab, they achieved nearly complete sequence coverage (100% for all but the Fd’ of adalimumab, which reached 97%) (#2.7 in Table 2), though without addressing the challenge of distinguishing between Ile and Leu residues. To mimic the analysis of antibodies with unknown sequences, they implemented a multi-step data processing approach: (i) identifying sequences *via* a database search, (ii) applying peptide *de novo* sequencing, and (iii) repeating the database search with the tentative sequence. The tentative sequences derived from bottom-up MS data were further validated through measured intact masses of the Fd’ and Lc subunits and MD MS data. In 2014, it was one of the first studies of this kind, and most of the data were processed manually.

In 2017, further exploring these strategies, Marshall and colleagues combined CID and ETD to analyze adalimumab’s Lc and Hc (65). These fragmentation events were carried out independently using an LTQ Velos Pro integrated with a custom-built 21 T FT-ICR MS. The combined results from eight targeted nano-LC FT-ICR MS/MS experiments, focusing on the 24+ charge state of the Lc and the 55+ charge state of the Hc, achieved an overall sequence coverage of 50%, with 81% coverage for the Lc and 38% for the Hc, all identified with a mass accuracy of better than 5 ppm (#2.11 in Table 2 and Fig. 3, Fig. 4, Fig. 5).

In 2021, Jinlan Zhang and colleagues optimized CID and ETD on a maXis II ETD UHR Q-TOF mass spectrometer for MD MS analysis of trastuzumab and adalimumab (66). Both antibodies were enzymatically cleaved using IdeS and reduced using TCEP to generate 25-kDa subunits. The sequence coverage achieved was 38% for trastuzumab and 37% for adalimumab (#2.20 in Table 2 and Fig. 3, Fig. 4, Fig. 5). In addition, major glycoforms were distinguished through intact native and denaturing MS analysis, with their relative abundances quantified.

In parallel, the Brodbelt group demonstrated the utility of UVPD fragmentation in 2016, particularly its complementarity to ETD (67). Using UVPD-equipped Orbitrap platforms they unambiguously localized the glycosylation site and characterized CDRs, providing an overall 60% sequence coverage on an IgG1 in a single LC-MS/MS run and up to 77% in combined four LC-MS/MS runs collected with varied laser parameters (67). Combining the results from UVPD and ETD techniques the sequence coverage reached 72% for adalimumab and 74% for trastuzumab, further supporting the use of multiple MS/MS methods (#2.8 in Table 2 and Fig. 3, Fig. 4, Fig. 5). Recently, sequence coverages of up to 97.1% for infliximab and 80.2% for cetuximab IgG1 were achieved using solely UVPD MS/MS on an Orbitrap Fusion Lumos instrument (68). In the latter study, Wang and colleagues improved sequence coverage by combining product ions generated from 13 separately isolated precursor charge states, each fragmented with UV pulse energy optimized for its charge state. These results demonstrate the benefit of combining product ions from multiple precursor charge states (#2.30 in Table 2 and Fig. 3, Fig. 4, Fig. 5). The main drawback of UVPD is the variety of product ions that can be formed (*a, b, c, x, y, z, w,* and *v-*types) (28), which complicates a confident assignment.

### Integrating TD and MD MS Workflows

In 2018, Kelleher's group combined three activation techniques: UVPD, ETD, and EThcD, for the MD-MS analysis of rituximab. Specifically, by employing TD MS analysis with UVPD and ETD, and MD MS on KGP or IdeS-generated subunits utilizing UVPD, ETD, and EThcD, they achieved a 90% sequence coverage (#1.8 in Table 1 and Fig. 3, Fig. 4, Fig. 5). Integrating TD and MD MS not only enabled comprehensive sequence coverage and PTM mapping comparable to bottom-up strategies but also provided more profound insights into rituximab's structure, including CDRs pairing, chain pairing, and intact mass, which are not accessible after extensive proteolysis (38). The confidence of these results was claimed to be ensured by maintaining a 10-ppm mass accuracy for product ion annotation, followed by isotopic fitting and manual curation of all the mismatched isotopic distributions.

In 2019, Ying Ge and colleagues conducted a comprehensive TD and MD-MS analysis of the SiLuLite mAb (Sigma mAb) (36). Using a high-resolution 12 T FT-ICR MS (SolariX XR, Bruker Daltonics), they performed a bird’s eye view analysis of all proteoforms, achieving isotopic resolution for all IgG1 proteoforms. The latter is particularly impressive considering the 150 kDa MW of these species. Subsequent single (isolated) charge state TD MS analysis, utilizing ECD and CID, enabled 23% sequence coverage annotation. MD MS analysis of 25 kDa subunits of the IgG1 antibody, also using ECD and CID, revealed key modifications, including C-terminal amidation, C-terminal glycine clipping, and proline amidation. Together, the TD and MD-MS analyses resulted in 76% sequence coverage (#1.10 in Table 1 and Fig. 3, Fig. 4, Fig. 5).

### Innovations in Instrumentation and Workflow Optimization

In 2018, Voinov’s group applied a hybrid ECD setup, combining UVPD and HCD (ECuvPD, EChcD) on a modified Orbitrap MS to analyze SiLuLite subunits. Antibody subunits were generated by IdeS digestion and disulfide bond reduction with DTT (69). The unique MS setup included an electromagnetostatic E x D cell (e-MSion Inc., Corvallis, Oregon) and a modified HCD cell enabling UVPD. ECD, ECuvPD, and EChcD were used to fragment the preselected 20+, 25+, and 24+ charge states of the Lc, Fc/2, and Fd subunits, respectively. All major types of product ions were formed and searched with 5 ppm mass accuracy. ECD and ECuvPD produced comparable results for each subunit and showed better performance than EChcD across all subunits. An overall sequence coverage of 86% was achieved in a single LC-MS/MS experiment (#2.14 in Table 2 and Fig. 3, Fig. 4, Fig. 5). As with UVPD, caution is advised when interpreting these results, given the inherent challenges in confidently assigning numerous product ion types. This MS setup was also used in TD/MD MS study of trastuzumab conducted by the Voinov and Shaw groups, mentioned in the TD MS chapter (#1.12 in Table 1 and Fig. 3, Fig. 4, Fig. 5) (43). Similarly, Stafford and colleagues demonstrated ECD-induced Lc cleavage from intact infliximab using an Agilent Q-TOF MS equipped with the E x D cell (70).

The field asymmetric ion mobility spectrometry (FAIMS) was added as a separation method for MD-MS of mAbs in 2019. Kelleher and colleagues utilized the FAIMS fractionation method to reduce the complexity of proteoform mass spectra and enhance the antibody sequence coverage (71). The reduced NIST mAb standard was analyzed using an Orbitrap Eclipse Tribrid equipped with FAIMS by applying multiple fragmentation techniques. A cumulative sequence coverage of approximately 50% was achieved through the application of HCD, CID, ETD, and UVPD dissociation methods on the Lc and HCD, CID, ETD, and EThcD on the Hc (#2.15 in Table 2 and Fig. 3, Fig. 4, Fig. 5). The annotation included all types of product ions: *c*- and *z*-ions, *b*- and *y*-ions, and *a*- and *x*-ions. Beyond sequence annotation, this study also identified the most abundant proteoforms observed in the NIST mAb standard, such as pyroglutamic acid and N-linked glycan G1F post-translational modifications.

One of the key limitations of dissociation methods in TD and MD-MS is the generation of charge-reduced but undissociated complexes, which congest the mass spectra, increase the baseline, and hinder product ion identification. Voinov’s and Shaw’s groups observed that conducting TD-MS analysis in native mode helps mitigate spectral congestion, as native antibody conformations adopt lower charge states (43). Alternatively, Brodbelt's group implemented gas-phase proton transfer charge reduction (PTCR) to reduce the charge states of product ions, thereby dispersing their distribution across the mass spectrum and improving the identification of antibody fragments (72). In the UVPD/PTCR MD-MS analysis performed by Brodbelt group, the precursor ion was first subjected to UVPD, after which a selected group of product ions was isolated, subjected to PTCR, and then analyzed in the Orbitrap mass analyzer. This process was iteratively applied to cover congested spectral regions, ultimately achieving 74% sequence coverage for the Lc of an ADC with a single payload (#2.17 in Table 2).

The benefits of the combined use of PTCR with CID and ETD MD MS analysis were also highlighted in a large interlaboratory study focused on mAb characterization (14). Notably, one of the most comprehensive sequence coverages was achieved, with 86% for the Lc and 66% for the Fd’ subunits of trastuzumab, and 76% for the Fd′ subunit of the NIST mAb by combining CID and ETD, both enhanced by PTCR (#2.18 in Table 2). These fragmentation reactions were performed in the linear ion trap followed by product ion detection in a high-resolution ICR cell of the 21 T LTQ FT-ICR MS platform with prior LC separation.

Neusüß and colleagues developed a two-dimensional fractionation strategy using capillary electrophoresis to reduce the complexity of mass spectra and effectively characterize minor fragments of the NIST mAb (73). Their analysis of the reduced antibody was conducted using an on-line CE(SDS)-CZE-MD MS platform, using HCD, ETD, and UVPD. As a result, they achieved an overall sequence coverage of 64% for the Lc of the NIST mAb (#2.22 in Table 2).

Chamot-Rooke’s group conducted approximately 200 MD MS experiments to determine the optimal combination of MS parameters, such as precursor ion charge state, activation technique and level (energy or time), and the number of technical replicates (74). They achieved 60% sequence coverage for both reduced NIST mAb and SiLuLite (Sigma mAb) by combining the results from their three best experiments, which included two EThcD experiments and one HCD experiment (#2.23 in Table 2 and Fig. 3, Fig. 4, Fig. 5). This work highlights the importance of selecting precursor ions in several charge states in MD MS analysis for two key reasons: i) targeting different precursor charge states can yield complementary sequence information by activating distinct fragmentation pathways; and ii) multiplex selection reduces the number of experiments required to obtain optimal sequence coverage.

Expanding instrumentation options, the Sciex and GSK teams demonstrated the effectiveness of the ZenoTOF 7600 (Sciex), a quadrupole TOF MS equipped with a proprietary ECD (EAD) cell, for the MD MS analysis of a biotinylated, goat-derived antihuman GSK mAb (75) (#2.24 in Table 2, Figs. 3, and 4). By combining CID and ECD techniques, they achieved 49% sequence coverage and established a linear dynamic range of 2 − 50 μg/ml, enabling accurate quantitation of the intact antibody spiked into serum.

One of the latest studies from Fornelli and colleagues set a new benchmark in the MD-MS antibody analysis, achieving a 94.2% sequence coverage of the NIST mAb (#2.25 in Table 2 and Fig. 3, Fig. 4, Fig. 5) (76). This work employed Orbitrap Eclipse (Thermo Fisher Scientific) equipped with an ETD/PTCR source and high mass range option to conduct the ETD, HCD, EThcD MS/MS, and corresponding PTCR-MS^3^ analysis. The MD MS method developed for the NIST mAb was validated across different antibody subclasses using SiLuLite mAbs (IgG1 and IgG4). This report highlighted three important aspects and developments in PTCR MD-MS. First, all MS1, MS/MS, and MS^3^ were recorded in the Orbitrap analyzer. Whereas the total ion charge remains similar between MS1 and MS/MS, the PTCR-based MS^3^ substantially reduces the number of charges by deprotonation. It is crucial to control the number of charges (ions) introduced into the Orbitrap, as space-charge effects—proportional to the number of charges injected - result in peak interference and mass shift. To address this, the researchers measured a total ion current (TIC) directly in the Orbitrap to estimate the actual number of charges. Second, the MS/MS and MS^3^ spectra were recorded at a high resolution of 240,000 (at *m/z* 200), allowing for the confident identification of ion clusters that were detected but unassigned at lower resolution, leading to increased sequence coverage. Third, spectral decongestion improved the confidence in ion matching, reduced assignment ambiguity, and minimized false positive product ion identifications.

In their subsequent study, Fornelli’s group applied this optimized MD MS workflow to ADCs, incorporating UVPD alongside the previously employed fragmentation techniques (77). The combination provided over 79.5% sequence coverage for Fd subunits carrying one or two payloads (#2.26 in Table 2).

## Beyond the Analysis of Purified Antibodies

Figure 5 provides a mAb-centric summary of sequence coverage values (%) reported in Tables 1, 2, Figures 3, and 4. Owing to advancements in TD and MD MS, the research community became better equipped to address complex biological questions. These include the analysis of antibodies in biological fluids, comprehensive proteoform profiling, PTM localization, and the characterization of emerging modalities, including BsAbs and ADCs. The studies conducted to date in these areas are reviewed in the following section.

### Analysis of ADCs

ADCs combine the precision of antibodies with the potency of cytotoxic drugs, allowing for targeted destruction of cancer cells while sparing healthy tissue. This targeted approach enhances treatment effectiveness and reduces systemic toxicity, making ADCs a promising class of therapeutics that need to be characterized with high precision (78).

In 2016, Gross’s group, in collaboration with researchers from Roche Group, analyzed structural aspects of the Fab-antigen complex using native TD-MS and denaturing MD-MS (#3.1 in Table 3) (79). The study confirmed a 2:2 binding stoichiometry between VEGF dimers and Fab-1, previously observed through X-ray crystallography. The locations of surface-exposed and flexible regions within the structure were identified through CID, ECD, and infrared multiple photon dissociation (IRMPD) MS/MS analyses. Specifically, CID was shown to facilitate desolvation, whereas ECD induced backbone cleavages to reveal flexible regions, and IRMPD generated product ions that remained bound in product ion complexes after ECD. The information obtained is valuable as a complement to X-ray crystallography, as flexible regions often complicate the crystallization process.

**Table 3:** Application of TD/MD/BU methods for MS analyses of mAbs in complex mixture or clinical samples

#	Application	mAb (antigen)	MS approaches	MS/MS method	Instrument	Sequence coverage	Ref.
3.1	Characterization of the stoichiometry of the Fab-antigen complex	Fab-1-VEGF	**Native MD**	CID, ECD, and IRMPD	12 T SolariX XR FT-ICR MS and	N/A	Zhang et al. 2016 (79)
			**MD** (DTT)		maXis 4G Q-TOF MS (Bruker Daltonics)		
3.2	Method development for analysis of IgGs spiked in human serum	Eculizumab, Rituximab, Vedolizumab, Infliximab, Adalimumab	**MD** IdeS	CID; ETD	21 T LTQ FT-ICR MS (NHMFL)	33–50% (Lc)	He et al. 2017 (65)
3.3	Method development for analysis of the endogenous mIgs from the serum of patients with AL amyloidosis or multiple myeloma	Endogenous mIgs	**MD** (IdeS + TCEP)	CID, ETD	21 T LTQ FT-ICR MS (NHMFL)	72% (Lc)	He et al. 2019 (93)
3.4	Characterization of the Igs from the serum of patients with systemic lupus erythematosus	12 standard Fabs Endogenous mIgs	**MD** (papain + TCEP)	CID, HCD, and ETD	LTQ Orbitrap Velos Pro (TFS)	67% (Lc) 40% (Fab)	Wang et al. 2019 (91)
3.5	Characterization of the drug conjugation sites on a site-specific ADC	CBW-03–106 DAR4	**MD** (IdeS + DTT)	HCD, ETD, UVPD	Orbitrap Fusion Lumos (TFS)	74%	Hernandez-Alba et al. 2019 (80)
3.6	Characterization of average DAR, positional isomers, conjugation sites, occupancy, and micro-variants for cysteine and lysine conjugated ADCs	Brentuximab -vedotin, Ado-trastuzumab – emtansine	**MD** (IdeS + DTT or KGP + DTT)	ETD	maXis II ETD Q-TOF MS (Bruker Daltonics)	35%	Chen et al. 2019 (81)
3.7	Characterization of DAR and drug conjugation sites for an IgG1-based ADC	IgG1 based (AbbVie)	**BU**	HCD	LTQ Orbitrap Elite (TFS)	97%	Watts et al. 2020 (82)
			**MD** (IdeZ + TCEP)	UVPD, ETD, EthcD	Orbitrap Fusion Lumos (TFS) equipped with a 193 nm excimer laser (Coherent ExciStar XS)	∼72% (Fab w/o payloads)	
3.8	Analysis of the antibody-antigen interaction related to the influenza virus	D1 H1–17/H3–14 IgG mAb and influenza A hemagglutinin (HA)	**Native TD** (amidase PNGase F)	HCD, UVPD	Q Exactive UHMR Orbitrap (TFS) equipped with a 193 nm ArF excimer laser (Coherent ExciStar XS)	UVPD – 42% (39% mAb & 49% HA1)	Mehaffey et al. 2020 (83)
3.9	Characterization of DAR, aa sequence and drug conjugation sites for cysteine-linked ADC	cysteine-linked ADC (AbbVie)	**MD** (DTT)	CID	12 T solariX XR FT-ICR MS (Bruker Daltonics)	52% (Lc0) (7 runs)	Larson et al. 2020 (84)
3.10	Characterization of DAR, aa sequence and drug conjugation sites for cysteine-linked ADC	NIST mAb cysteine-linked ADC (AbbVie)	**nTD**	CID (MS^2^ and MS^3^)	timsTOF Pro (Bruker Daltonics)	6.1% (Lc)	Larson et al. 2021 (85)
3.11	Characterization of the Fab (IgG1) from plasma of healthy donors and septic patients	Endogenous IgG1	**MD and BU** (IgdE and TCEP)	HCD or ETD	Orbitrap Fusion Lumos and Q Exactive HF-X Orbitrap (TFS)	100% (Fab) (BU & MD)	Bondt et al. 2021 (94)
3.12	Characterization of the Lcs (IgGs) from the urine of patients with multiple myeloma	Endogenous Lc antibodies	**TD and BU** (with/without TCEP)	HCD, CID, EthcD, and UVPD	Q Exactive Plus and Orbitrap Fusion Lumos (TFS)	MD – 89% (Lc) – 12 runs BU – 100% (Lc)	Dupre et al. 2021 (96)
3.13	Characterization of M-protein in the serum of kidney transplant patients	M-protein	**MD and BU** (IgdE + TCEP)	BU – HCD and EthcD; MD – EthcD	Orbitrap Fusion or Lumos (TFS)	43% (Fab)	Peng et al. 2023 (95)
3.14	Conducting pharmacokinetic studies of pembrolizumab spiked in mouse plasma	Pembrolizumab	**MD** (PNGase F, IdeS or IdeS + TCEP)	HCD or EthcD	Orbitrap Eclipse and Orbitrap Exploris 480 (TFS)	55% (with Eclipse at 100 μg/ml)	Dhenin, Lafont, et al. 2023 (92)
3.15	Method development for ADC analysis, including sequencing and characterization of drug conjugation sites	Trastuzumab deruxtecan	**MD** (IdeS + DTT)	CID, ETD, EthcD with PTCR	Orbitrap Eclipse (TFS)	100% (all methods + internal product ions) 50% (EthcD with PTCR)	Beaumal et al. 2024 (86)
3.16	Characterization of aa sequence and drug conjugation sites for single-domain ADC (sdADC)	anti EGFR sdADC	**MD** (DTT)	ETD, HCD, and 213 nm UVPD	Orbitrap Eclipse (TFS)	87% (sdADC)	Benazza et al. 2024 (87)
3.17	Glycosylation profiling and structural analysis of glycans of glycoengineered mAb	Glycoengineered SiLuLite, trastuzumab and rituximab	**TD**	MALDI-ISD, CID	15 T solariX 2xR FT-ICR MS equipped with a CombiSource and a ParaCell (Bruker Daltonics)	MS/MS glycan release	Senini et al. 2024 (90)
3.18	Glycosylation and glycoproteoform profiling	recombinant IgA1	**Native TD and BU**	BU (HCD, EThcD) Native (ECCR)	Q Exactive UHMR Orbitrap (TFS)	Glycoproteoform characterization	Peris-Diaz et al. 2024 (89)
3.19	Payload quantification	ADC2 DAR4, ADC2 DAR8, trastuzumab emtansine	**MD**	CID	ZenoTOF 7600 (Sciex)	Payload release	Yuan et al. 2024 (88)

Definitions and abbreviations are as in Table 1.

Important features to characterize in ADCs are the drug-to-antibody ratios (DARs) as well as the payload location (if possible). In 2019 Cianferani and colleagues analyzed DAR4 ADC complexes using MD-MS on the Orbitrap platform combining HCD, ETD, and UVPD (80). Their study demonstrated that UVPD, when used as a standalone method, outperformed both HCD and ETD, not only in terms of overall sequence coverage but also in the identification of drug and glycosylation binding sites. By integrating the results from these three activation techniques, the overall sequence coverage of the ADC reached 74% (#3.5 in Table 3).

In the same 2019, the Ge group employed MD-MS with ETD-enabled Q-TOF MS to assess multiple quality attributes of two ADC variants: cysteine-conjugated brentuximab vedotin (BV) and lysine-conjugated ado-trastuzumab emtansine (T-DM1) (81). The ADCs were digested with IdeS and subjected to disulfide bond reduction, generating seven distinct subunits: Fc/2, Lc without drug (Lc0), Lc with one drug (Lc1), and Fd’ subunits conjugated with 0 to three drug molecules (Fd’0–Fd’3). The average DAR values were calculated as 4.0 for BV and 3.5 for T-DM1. Sequence coverage for the fully reduced subunits was approximately 35% (#3.6 in Table 3). Given the small size of the drugs relative to the antibody subunits, similar ionization efficiency was observed between conjugated and unconjugated forms, enabling accurate calculation of the relative proportions of drug-conjugated subunits. Additionally, ETD applied to partially reduced subunits enabled the resolution of positional isomers, allowing precise localization of conjugation sites and determination of their relative abundance.

The group of Brodbelt, in collaboration with AbbVie, also studied IgG1-based ADC using MD-MS by combining UVPD, ETD, and EThcD techniques (82). While bottom-up MS analysis verified 97% of mAb sequence coverage (#3.7 in Table 3), it provided limited information about payload sites, with a few peptides containing payload identified. The MD MS analysis of seven ADC subunits obtained after digestion with IdeZ (a cysteine enzyme that cleaves mouse IgG2 and IgG3 below the hinge region) and reduction of disulfide bonds, yielded 74% sequence coverage. Among the methods, UVPD was found to generate the highest number of unique product ions, whereas ETD and EThcD yielded complementary product ions essential for accurate payload site determination, particularly in subunits with heterogeneous payload distribution. C-terminal ions carrying the payload were critical for confirming its location. Yet, the abundance of these ions decreased as more payloads were added to the subunits, likely due to truncation of the payload during MS/MS analysis, leading to unassignable product ions. Thus, combining these complementary activation methods produced a diverse set of C-terminal ions, facilitating the localization of payload conjugation sites.

Extending their exploration of antibody applications, Brodbelt’s group also utilized native TD MS to investigate antibody-antigen interactions, combining HCD and UVPD to analyze the D1 H1–17/H3–14 antibody, which targets hemagglutinin (HA), a key antigen of the influenza virus (83). As expected, UVPD significantly outperformed HCD in sequence coverage, achieving 60% compared to 27% (#3.8 in Table 3). When analyzing the Ab·2HA1 complex, both activation methods produced lower sequence coverage for HA1 compared to its unbound form. Nevertheless, the presence of HA did not markedly affect sequence coverage of either antibody chain. Notably, UVPD produced only *b*- and *y*-type ions within the epitope regions of the bound complex, whereas in the unbound antibody, a more diverse array of fragment ions - including *a*/*x*, *b*/*y* and *c*/*z* types - was observed.

Ge's group, also in collaboration with AbbVie, conducted a series of studies on cysteine-linked ADCs using both MD and TD-MS approaches. In one study, they applied CID-based MD-MS to reduced ADCs, using a 12 T FT-ICR MS (SolariX XR, Bruker Daltonics), and combined product ions from seven experiments with varying collision energies to achieve 52% sequence coverage of the unconjugated light chain (Lc0) (#3.9 in Table 3) (84). Within a one-hour analysis time, approach provided information about DAR, primary sequence, and localization of drug conjugation sites.

In a subsequent study, the Ge team employed native TD-MS using a high-resolution trapped ion mobility spectrometry TOF MS (timsTOF Pro, Bruker Daltonics) to rapidly assess key ADC quality attributes (85). Within only10 min of direct injection, ion mobility separation effectively resolved all DAR species, facilitated calculation of average DAR values, and detected low-abundance species such as DAR eight in the MS1 scan. Notably, the study revealed conformational changes induced by drug conjugation: the ADC structure became more compact compared to the unconjugated mAb, and higher drug loads correlated with increased collision cross section values. To further dissect structural details, an isCID-based MS/MS approach was used to dissociate non-covalently linked subunits, followed by CID-based MS^3^ on the isolated Lc with one payload (Lc1), achieving 6.1% residue cleavage (#3.10 in Table 3). These results highlight the potential of MS^3^–based approaches to perform deep sequencing of the ADC and represent a promising direction for future TD/MD MS method development.

### Advanced Workflows and Emerging ADC Modalities

Further advancing the field, Hernandez-Alba and colleagues applied TD and MD MS to study both conventional and single-domain ADCs (sdADCs). Their first investigation focused on trastuzumab deruxtecan, a therapeutic site-specific ADC (86). The team integrated intact MS with advanced MD MS techniques, including PTCR and internal product ion analysis. Adding PTCR after subunit fragmentation improved spectral clarity and enhanced product ion detection in congested regions by 1.5- to 2.5-fold, leading to a sequence coverage increase of 2% to 13% and an overall coverage of up to 87% (#3.15 in Table 3). Whereas internal product ion analysis presumably allowed full sequencing of the Fc/2, Lc, and Fd’ subunits, it provided limited contribution to localizing the payload attachment, which was situated at the C-terminus.

Their second study focused on sdADCs—compact therapeutic constructs derived from Fd’ subunits (87). Both TD and MD MS analyses were conducted using HCD, ETD, and UVPD. For non-reduced sdADCs, the sequence coverage was approximately 33%, whereas reduction of disulfide bonds improved sequence coverage to 85% (#3.16 in Table 3). In this instance, HCD was particularly informative for identifying the conjugation site, outperforming ETD and UVPD. This advantage was attributed to the C-terminal location of the conjugation site, which is easily accessible to HCD, and to the preservation of the attached drug molecule during fragmentation.

Finally, Rosenbaum and colleagues demonstrated the application of MD MS for analyzing payload release from ADCs spiked into plasma samples (88). Using a digestion-free workflow on a 7600 ZenoTOF MS (Sciex), they employed CID to selectively dissociate the payload by applying optimized collision energies. This approach enabled quantification of ADCs based on characteristic MS/MS product ions derived from the payload (#3.19 in Table 3). Notably, the method proved effective for a range of linker-payload designs, including ADCs with non-cleavable linkers such as trastuzumab emtansine, underscoring its versatility for accurate ADC quantification in complex biological matrices.

### Novel Approaches to Glycosylation Analysis

The Heck group conducted a comprehensive analysis of the N- and O-glycosylation sites and glycoproteoforms of recombinant IgA1, an antibody characterized by extensive glycan heterogeneity due to multiple glycosylation sites (#3.18 in Table 3) (89). To achieve this, they applied both (glyco)peptide-centric and protein-centric mass spectrometry workflows, enabling the assignment of individual N- and O-glycosylation sites and the characterization of intact glycoproteoforms. In addition to classical bottom-up MS profiling, native MS was used to assess the intact glycoproteins, revealing highly congested mass spectra due to glycoform heterogeneity. To address this spectral complexity, native top-down MS employing electron-capture charge reduction (ECCR) was implemented. In ECCR, highly charged proteins interact with free electrons without undergoing fragmentation, effectively reducing charge states and shifting ions to higher *m/z* ranges - functionally analogous to PTCR. By performing “sliced” ECCR experiments, which involve sequential selection of narrow *m/z* windows across the native mass spectrum, the researchers identified asymmetrical N-glycan occupancy across the IgA1 molecule.

In 2024, Nicolardi and colleagues performed a detailed profiling of a glycoengineered mAb using TD MS with MALDI-ISD on a 15 T FT-ICR MS (90). The key innovation of this work is the direct characterization of glycosylation without the need for preliminary enzymatic glycan release. The MALDI matrix containing sodium selectively suppressed signals from product ions, which are more effectively detected as protonated ions rather than sodiated ions. This suppression improved the detection of glycans removed from the mAbs by ion activation and dissociation. The method allowed for identifying low-abundance glycoforms, such as the Man5/Man5 variants in SiLuLite mAb and rituximab, which are typically challenging to detect (#3.17 in Table 3). Notably, while these minor glycoforms had been previously detected by intact mass analysis, that approach required signal averaging of unreduced Orbitrap transients across multiple LC-MS runs (14). Additionally, Nicolardi and colleagues utilized CID to offer insights into the monosaccharide composition of the selected glycans enhancing the depth of glycosylation analysis.

### Antibody Mixture Analysis

An ETD-based MD-MS analysis of two simple antibody mixtures using an LTQ Orbitrap Elite instrument was reported by Tsybin’s group in 2014, later peer-reviewed in 2022 (17). Both mixtures were compiled of 50 kDa Fab subunits obtained by papain digestion: one from a two-antibody combination (IgG1 trastuzumab and rituximab), and another from a three-antibody set comprising trastuzumab (IgG1), adalimumab (IgG1), and natalizumab (IgG4). The study demonstrated high complexity of antibody mixture analysis, even at their subunit level and with only a few subunits present in the mixture. Indeed, baseline separation of the three Fab subunits with RPLC was not achieved, presumably due to their similar hydrophobic properties. Nevertheless, it was possible to isolate a group of three charge states per Fab, enabling the acquisition of good quality ETD MS/MS data. Although sequence coverage was limited, around 15% for both the Lc and Fd subunits forming the Fab, the results were sufficient to detect internal product ions indicative of heavy–light chain pairing. In case of trastuzumab and rituximab, co-elution prevented isolation of individual charge states. Nevertheless, ETD-based MD MS following co-isolation of several charge states from both Fabs resulted in unique product ions that covered the CDR3, supporting chain pairing analysis *via* internal (branched) product ions.

In 2017, Marshall and colleagues successfully identified five antibodies spiked in a serum sample (65). The MD MS analyses were performed using an LC system coupled with a custom-built 21 T FT-ICR mass spectrometer (NHMFL) with fragmentation by CID and ETD. The combined product ions resulted in Lc sequence coverages of 33%, 37%, 42%, 47%, and 50% for eculizumab, rituximab, vedolizumab, infliximab, and adalimumab, respectively (#3.2 in Table 3). Interestingly, the data showed greater mass error for product ions than precursor ions, with values ranging from 0.2 to 0.4 ppm for precursor ions compared to 2.9 to 4.7 ppm for product ions. Further analysis indicated that deconvolution introduced a 3- to 5-fold increase in mass error, highlighting a limitation of some current deconvolution algorithms—particularly those based on the averagine model.

Extending the application of TD MS to more complex mixtures, Wu and colleagues optimized an ultra-high-pressure (UHP)LC TD MS platform and applied it to analyze a mixture of 12 standard monoclonal antibody Fab fragments (91). The combination of CID, HCD, and ETD methods resulted in an average sequence coverage of 67% for the Lc and 40% for the Fd’ subunits (#3.4 in Table 3). The confidence of these product ion annotations was manually verified and confirmed. This optimized (UHP)LC TD MS platform was subsequently employed to characterize auto-antibodies in human serum from a patient with systemic lupus erythematosus, successfully identifying 47 unique light chains and 16 Fd’ subunits.

In collaboration with Sanofi, Chamot-Rooke's group addressed another problem in antibody analysis, such as the *in vivo* stability of antibodies (92). To achieve this, pharmacokinetics studies were conducted by spiking pembrolizumab into mouse plasma at concentrations ranging from 0.5 to 100 μg/ml, followed by automated immunocapture and analysis using TD and MD MS. Two cutting-edge Orbitrap platforms, the tribrid Orbitrap Eclipse and Orbitrap Exploris 480, were employed for the study. At a 100 μg/ml concentration, a maximum sequence coverage of 55% for pembrolizumab was achieved using the Orbitrap Eclipse (#3.14 in Table 3). Overall, the ETD-equipped Eclipse outperformed the HCD-equipped Exploris 480 in sequence coverage (62% vs. 28%), primarily due to the contributions of EThcD. However, sequence coverage decreased more rapidly with decreasing pembrolizumab concentration in the Orbitrap Eclipse platform, yielding exploitable results only down to 5 μg/ml. In contrast, HCD-based Exploris 480 analysis was less sensitive to antibody concentration, maintaining performance at lower concentrations.

### Antibody Repertoire Analysis

One of the first MD MS studies of antibody repertoire was reported by the Marshall group in 2019 (#3.3 in Table 3). It focused on analyzing endogenous antibodies in human serum samples by employing an LC system coupled with a custom-built 21 T FT-ICR MS (NHMFL) with fragmentation by CID and ETD (93). The main challenge was the reliable identification of antibody sequences in the absence of corresponding database entries, particularly those associated with diseases like amyloidosis and multiple myeloma. To address this, the authors developed a database-aided *de novo* sequencing approach. This approach first identified homologous antibody regions through database searches, then applied *de novo* sequencing to determine the short variable regions. This was achieved by comparing observed mass differences to an accurate mass database of 1 to 4 amino acid residues, using a 10-ppm mass error threshold.

In 2021, Heck and colleagues conducted a detailed qualitative and quantitative analysis of Fab subunits from endogenous IgG1 antibodies in human plasma from healthy donors and septic patients (94). Despite the immense theoretical diversity of human immunoglobulins, presumably extending beyond 10^15^, the study identified between 35 and 543 distinct IgG1 clones per sample, with a median of 196 dominant clones. For quantification, two monoclonal IgG1 antibodies at known concentrations were spiked into each sample. This revealed that the top 30 most abundant clones accounted for a median 71.8% of the total IgG1 molecules detected. The analysis of the most abundant clone was carried out using both bottom-up MS and MD-MS approaches. The bottom-up MS alone was insufficient for full sequence identification due to interference from co-isolated peptides derived from other plasma clones. Thus, researchers iteratively extended the sequence information obtained from the MD-MS approach with the *de novo*-identified peptides from the bottom-up MS approach. This integrative approach enabled complete sequence coverage of the endogenous IgG1 clone (#3.11 in Table 3).

This landmark study on antibody analysis using MS methods was followed by the analysis of M-protein (antibody) sequences directly from the blood of kidney transplant patients (95). Similar to the previous research, peptide *de novo* sequencing from bottom-up MS was validated using native MS and MD LC-MS/MS. EThcD-based MD-MS analysis of the Lc and Fd’ subunits yielded 43% sequence coverage (#3.13 in Table 3). During the Fab analysis, an unusual antibody profile was discovered in one of the patient samples: the IgG1 repertoire was dominated by a small number of highly abundant clones with elevated molecular masses. MS data provided detailed information into the glycosylation of the Fab region, including both the site and composition of glycan modifications on the Hc of the M-protein. Based on these data, combined with clinical assays such as serum immunofixation, a diagnosis of monoclonal gammopathy of undetermined significance was made, potentially avoiding the need for a bone marrow biopsy.

During the same 2021, Chamot-Rooke and colleagues applied a combined MD and bottom-up MS methods to comprehensively annotate antibody’s Lc from patients with multiple myeloma (96). In this disease, B-cells produce large amounts of monoclonal Lcs and intact IgGs, resulting in a highly diverse Lc repertoire driven by somatic recombination and various mutations. The combined MD-MS and bottom-up MS-based workflow began with intact mass profiling, peptide-level *de novo* sequencing using multiple enzymes and bottom-up MS. Subsequent MD-MS analysis of disulfide-reduced subunits was used to define proteoform structures, which were further validated by bottom-up MS, including differentiation between Ile and Leu amino acids. MD MS analysis performed on an Orbitrap Eclipse platform, integrating results from multiple fragmentation methods (HCD, CID, EThcD, and UVPD), enabled the researchers to achieve an 89% sequence coverage and identify 470 product ions (#3.12 in Table 3). The authors reported the discovery of a novel PTM, which appeared to result from the patient's treatment with a small-molecule chemotherapy adjuvant. Overall, 10 different Lc antibodies were characterized, demonstrating the effectiveness of the workflow.

## Overview and Outlook

This comprehensive review of TD and MD-MS for antibody analysis provides an up-to-date overview of the literature and highlights the remarkable progress achieved over the past 30 years. The following sections first overview the key technical and methodological advancements that have shaped the field. This is followed by an outlook on emerging trends in sample preparation, instrumentation, ion activation and fragmentation strategies, and data analysis. While not exhaustive, this outlook presents selected examples that point toward promising directions for the continued evolution of TD/MD MS in antibody characterization.

### Progress in TD/MD MS for Antibody Characterization

From an analyte perspective, the majority of studies have focused on IgG1 as a model antibody, owing to its widespread use in therapeutic applications and well-characterized structure, Figure 5. To date, TD/MD MS analyses with reported sequence coverage have been described in the peer-reviewed literature for the following IgGs: eight IgG1s (adalimumab, bevacizumab, cetuximab, infliximab, NIST mAb, rituximab, SiLuLite, and trastuzumab), one IgG2 (panitumumab), and two IgG4s (natalizumab and SiLuLite K4). Among these, trastuzumab has been the most extensively studied antibody with TD/MD MS, followed by adalimumab and the NIST mAb.

Sequence coverage remains the primary metric for evaluating method performance in TD/MD MS, as it reflects both the analytical complexity of the approach and the challenges associated with PTMs characterization, an aspect that could, in principle, serve as an alternative performance metric. Figures 3 and 4 illustrate the progression of TD/MD MS techniques over time, highlighting improvements in sequence coverage as a key indicator of methodological advancement. This progress is largely driven by the choice of MS/MS methods and their strategic application, such as the combination of complementary fragmentation techniques. Notably, electron-based dissociation methods, particularly ECD and ETD, have served as the foundation for TD/MD MS of antibodies. More recently, UVPD has enabled some of the most comprehensive sequencing results; however, the diversity of fragmentation pathways in UVPD necessitates careful validation of product ion assignments. Similarly, incorporating internal product ions into data analysis workflows has significantly enhanced TD/MD MS output but also requires meticulous assignment and validation. Currently, the most advanced methodologies integrate multiple MS/MS techniques with gas-phase charge reduction strategies, including ion–ion proton transfer charge reduction (PTCR) and ion–electron charge compensation reactions (ECCR) (97, 98), to further improve spectral clarity and sequence coverage.

In addition to sequence coverage and MS/MS methods, Tables 1 and 2 provide further details on the experimental workflows and techniques employed in each study. To date, the Orbitrap has been the mass spectrometer of choice for TD/MD MS of antibodies, featuring in over 60% of published studies. TOF MS and FT-ICR MS platforms have each been employed in approximately 20% of the studies. Most TD/MD MS analyses have been performed using ESI, typically *via* direct infusion under denaturing or native conditions. However, several studies have incorporated on-line separation techniques with ESI, such as micro- or nano-flow liquid LC, and, to a lesser extent, CZE. MALDI offers an attractive alternative, primarily due to its ISD capabilities, but its use in this context remains relatively limited.

TD/MD MS now supports analysis of several PTMs, such as N-terminal pyroglutamate formation and C-terminal lysine/glycine clipping, using terminal product ions (29, 40, 51, 52). Disulfide bond mapping remains difficult, although fragmentation efficiency has been used to infer connectivity, as demonstrated in ETD-based TD MS (37) and AI-ETD studies using variable laser power and reaction times (41). More precise localization has been achieved *via* internal product ions (46) or electrochemical reduction coupled with high-resolution 15 T FT-ICR MS (48).

Oxidation detection and localization have been demonstrated using TD MS with in-source CID (pseudo-MS^3^) (29), and ETD-based MD MS in forced oxidation studies, particularly for methionine (52) and arginine residues (51). N-glycosylation has been detected in TD MS using AI-ETD (41) and internal product ions (46), albeit without precise site localization. In contrast, MD-MS studies have enabled both identification and localization of glycosylation sites using ETD (52), HCD with online CZE separation (51), and hybrid fragmentation techniques such as UVPD-ETD (67), CID-ETD (66), and ECD-ECuvPD-EChcD (69).

Although a broad range of applications has been demonstrated (Table 3), most of these studies have been conducted in just the past 5 years. Notably, method development has been closely linked to application-driven research, such as the analysis of complex antibody mixtures and non-standard modalities, underscoring the field’s rapid evolution. These advancements have been made possible by strong collaborations between academic institutions and biopharmaceutical industry leaders, whose combined efforts have created a synergistic effect that continues to drive innovation across the field.

### Trends in Sample Preparation

Current and future developments in sample preparation for intact protein analysis and TD/MD MS have been reviewed elsewhere (99). Antibody-specific advancements include several structure-specific enzymes that specifically cleave various classes of antibodies. The recently introduced enzymes tend to complement the well-established IdeS and KGP-based workflows and to facilitate subunit separation and PTMs detection. These newer enzymes promise improved performance in mAb analysis by refining digestion workflows and reducing artifacts. Advances have also been made in chromatographic strategies tailored for antibody analysis. For instance, the integration of online hydrophobic interaction chromatography (HIC) with mass spectrometry has enabled efficient separation of intact antibodies and their multimers, while preserving non-covalent interactions (100). This development facilitates antibody complexes characterization under native-like conditions. In addition, the automated sample preparation, *e.g.*, using the SampleStream technology (Integrated Protein Technologies Inc) (101), aims to integrate multiple steps (digestion and buffer exchange, including chemical reduction) into an automated, continuous-flow system. These recent innovations aim to minimize sample loss and contamination and increase throughput and reliability, enabling a direct connection between sample preparation and MS, which is especially useful for large-scale mAb analysis.

### Trends in MS Instrumentation

Advances in MS instrumentation continue to drive progress in TD and MD antibody MS analysis. This is demonstrated by the highest reported 95% coverage of trastuzumab obtained using MD MS on the Orbitrap Ascend (#2.27 in Table 2 and Fig. 3) and the remarkable 64% sequence coverage achieved by HCD on the state-of-the-art Orbitrap Astral of three various antibodies (#2.28 in Table 2 and Fig. 3) (102, 103). The Orbitrap Ascend, particularly in its Structural Biology configuration, is arguably the most powerful and versatile commercially available instrument for TD/MD MS, offering ultra-high resolution, a wide mass range, and flexible multimodal MS/MS capabilities.

Currently, the two flagship FT-ICR MS instruments are a 21 T FT-ICR MS system interfaced to an Orbitrap Eclipse at NHMFL and a 21 T FT-ICR MS system interfaced to an Orbitrap Exploris 480 at PNNL. Both systems provide exceptional performance in TD/MD MS and are open to external users. The only FT-ICR MS manufacturing company, Bruker Daltonics, continues to enhance the performance of their commercial systems specifically for TD/MD MS, with the current developments aiming to integrate ion mobility, *e.g.*, tims technology (104), into a single powerful platform.

Despite the exceptional performance of current ICR and Orbitrap FTMS platforms, there is still room for improvement in extracting maximal information from their time-domain ion signals (transients) through advanced real-time and post-acquisition processing. This potential is underscored by the demonstrated value of high-performance data acquisition systems interfaced with Orbitrap instruments (105). Post-processing approaches, such as transient averaging or selection of optimal transient lengths (78, 106), along with other strategies aimed at enhancing data quality, can significantly improve signal-to-noise ratios and resolution. Similar benefits have been observed on FT-ICR MS platforms, where external high-performance acquisition and processing systems, such as FTMS Boosters (Spectroswiss), interfaced with a 15 T FT-ICR MS (SolariX, Bruker Daltonics), have enhanced intact mass analysis of antibodies (107).

FTMS time-domain ion signals (transients) also open the door to novel ion measurement strategies, such as individual ion counting or charge detection mass spectrometry (CDMS) (108, 109, 110). In particular, Orbitrap-based CDMS offers promising capabilities for enhancing the performance of both intact mass analysis and TD/MD MS (110, 111). These advances apply not only to antibodies but also to larger biotherapeutics such as adeno-associated viruses (AAVs). AAVs consist of three viral proteins, VP1, VP2, and VP3, which range in molecular weight from approximately 60 to 80 kDa. Fornelli and colleagues applied conventional TD MS to these VP proteins using various MS/MS techniques, achieving sequence coverages of up to 40% per protein (112).

Among recent innovations in TOF MS, the high-resolution timsTOF, equipped with the multimodal MS^n^ capabilities of the integrated Omnitrap (113), is being advanced by Bruker Daltonics with a particular focus on TD/MD MS applications and is a much welcome advancement. Implementation of the Omnitrap on a high-resolution TOF MS system will expand the current offer of ECD-enabled TOF MS instruments that include a ZenoTOF MS from Sciex (55) and a 6545XT AdvanceBio LC/Q-TOF from Agilent (70) and a SELECT SERIES Cyclic IMS TOF MS from Waters (114, 115). However, the resolution currently achievable with conventional TOF MS technology (typically up to ∼100,000) may limit its utility in advanced TD/MD analyses of large proteins, including antibodies. As a result, emerging higher-resolution TOF MS instruments based on multiple ion reflections (MRT platforms), which offer resolution exceeding 100,000 across a wide mass range (116, 117, 118), are expected to become even more powerful additions to the TD/MD-MS platform landscape.

### Trends in Dissociation Techniques

In gas-phase techniques, developments in MS^3^ and MS^4^ approaches, which are built on sequential ion activation and dissociation schemes, offer more detailed structural insights by isolating and fragmenting ions through multiple stages (119). This increases the depth of structural information captured, especially for complex proteoforms, and enhances annotation accuracy, especially for internal product ions. The increasing use of PTCR combined with MS/MS methods, including in MS^3^ and MS^4^ formats, appears to be one of the main avenues for further progress in TD/MD MS development and applications. Recent studies achieved 94.2% and 95% antibody sequence coverage employing MD MS analysis with PTCR-MS^3^ on Orbitrap Eclipse and Orbitrap Ascend, respectively (#2.25 and #2.27 in Table 2 and Fig. 3) (76, 102). The AI-ETD-based TD MS result from Coon's group encourages current developments in the field, including the implementation of the AI-ECD approach in the Omnitrap-Orbitrap platform aiming at high-performance TD MS of proteins in mAbs subunits mass range (120). Even without supplemental ion activation with photons, the optimized Omnitrap–Orbitrap–FTMS Booster platform enabled comprehensive MD MS sequencing on a chromatographic time scale, as demonstrated in the analysis of a multiple myeloma light chain (#2.29 in Table 2) (121), achieving an exceptional ∼93% sequence coverage in a single LC-MS/MS run.

### Trends in Data Analysis

On the data analysis side, the progress applies to all types of analytes and is not specific to antibody analysis. It is, however, probable that dedicated software tools for mAb analysis would significantly advance the field. Software advancements have driven the transition from manual to more automated processing, significantly improving the accessibility, throughput, and efficiency of TD and MD-MS data analysis. Open-source tools such as ProSight Lite (122), TopPIC Suite (123), ClipsMS (124), and MASH Suite Pro (125), as well as commercial implementations of TD/MD MS workflows in ProSight PC and TDValidator (Proteinaceous), Expressionist (Genedata), OmniScape (Bruker Daltonics), BioPharma Finder (Thermo Fisher Scientific), MassHunter BioConfirm (Agilent), BioPharmaView (Sciex), Intact Mass (Protein Metrics), AutoVectis (VibratIon), Peak-by-Peak BioPharma (Spectroswiss), and others are all critical and complementary, as they differ in the underlying algorithms and methodology.

Deconvolution methods continue to evolve, however, with most studies relying on a single algorithm. Presently, there is a trend toward integrating multiple deconvolution approaches to enhance confidence in data interpretation (126). At the same time, there is a noticeable shift in data analysis from mass spectra deconvolution to direct product ion isotopic distribution matching between the experimental and theoretical (simulated) datasets (127). Even though the validation of product ions involves stringent criteria, including mass tolerance distribution centered at 0 ppm with a standard deviation of 5 ppm, manual validation is still required to improve the reliability of the results. The latter remains challenging and not standardized. The analysis of internal product ions is gaining attention with several tools currently in development (128). However, despite their potential, internal ions annotation should be done with caution due to the vast number of theoretically possible candidates, which significantly increases the risk of false-positive product ion assignments (128). Additionally, improvements in spectral quality, particularly through improved ion detector sensitivity and higher-throughput mass analyzers, are complemented by enhanced computational abilities of modern tools. The latter can handle increasingly large sizes of mass spectral datasets, thus eliminating the need for extensive data reduction prior to data processing and analysis. For example, direct processing of transients or mass spectra acquired in full profile in Orbitrap-based TD/MD MS has proven to enhance sensitivity and annotate low-abundant product ions (50).

The enhanced spectral quality is particularly important when attempting *de novo* sequencing of antibodies in their intact or subunit form. Missing cleavage sites and relatively high mass errors may prohibit the progression of *de novo* sequencing algorithms (129). Therefore, it is not surprising that up to now only three studies have attempted protein-level *de novo* antibody sequencing, all of which remain either partial or rely on database-assisted searches (53, 56, 93). Significant advancements are still needed to automate protein-level MS *de novo* sequencing of antibodies (130, 131).

Finally, to tackle the challenges and limitations of the conventional sequencing approaches, other data analysis methods have been proposed specifically for TD/MD MS. For example, the All-Ion Differential Analysis (AIDA) method, which compares all product ions across different sample conditions (unmodified and variant) before spectral annotation, has been proposed as an alternative to classical TD/MD MS analysis (132, 133). The authors suggest that AIDA enhances TD/MD MS by characterizing sequence variations with higher sensitivity than conventional product ion annotation, enabling precise localization of modifications such as isomerization, deamidation, and oxidation in mAbs. However, they also note that AIDA may be limited by non-fragmented antibody regions and challenges in resolving multiple concomitant modifications, which could introduce biases in data interpretation (132).

### Consortium for Top-Down Proteomics—A Collaborative Effort

**Fig. 6:**
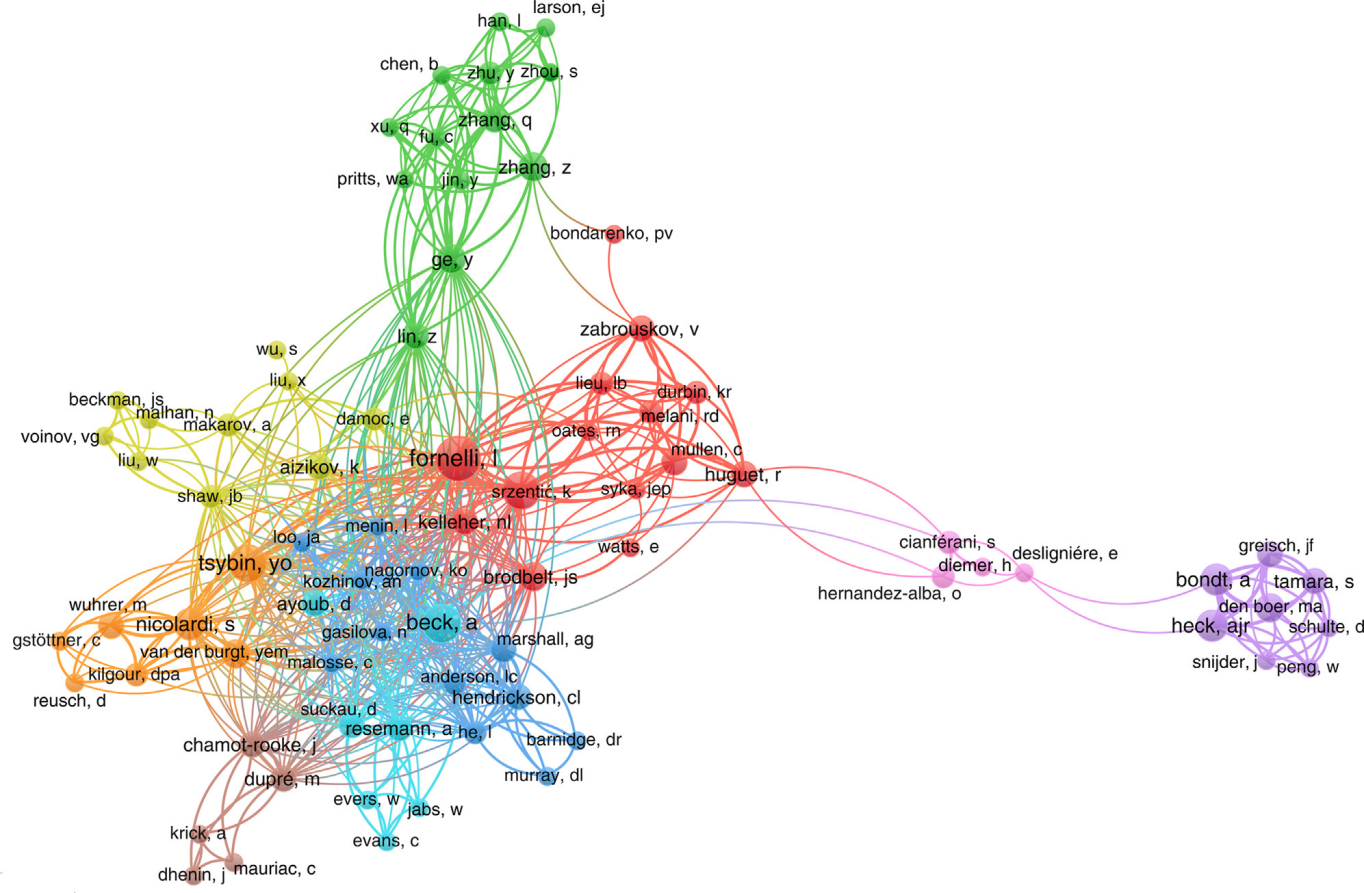
**Research network graph generated based on co-authorship in TD and MD MS antibody analysis peer-reviewed publications.** Each node symbolizes an individual researcher, with connecting lines indicating co-authorship links; stronger connections correspond to more frequent collaborations. A total of 62 research papers (listed in Table 1, Table 2, Table 3) involving 319 authors were analyzed, with 80 authors contributing to at least two papers on TD and MD MS antibody analysis. Distinct colors denote clusters of closely collaborating researchers. This network was generated using VOSviewer version 1.6.20 (138).

The Consortium for Top-Down Proteomics, or CTDP (https://ctdp.org), leads efforts in standardizing protocols, ensuring that TD and MD MS approaches are robust and reproducible across different platforms (14, 134). Figure 6 describes the network of researchers involved in TD/MD MS studies and quantifies the interaction with other members from the TD/MD MS community. Most of the researchers affiliated with CTDP are part of the largest network in the center of the visualization. Collaborative efforts like these create and offer access to large TD/MD MS databases and thus will be pivotal for integrating machine learning algorithms into data processing and further optimizing the most challenging algorithms, such as *de novo* sequencing.

Looking ahead, combining advancements in sample preparation, gas-phase methodologies, and data processing algorithms will significantly enhance the applicability of TD and MD MS in therapeutic antibody characterization in the biopharmaceutical industry and their acceptance among the regulatory agencies. Moreover, the increasing diversity and complexity of next-generation biotherapeutics can make them more prone to *in vivo* biotransformation than regular mAbs, which can impact both efficacy and safety. Biotransformation products are therefore now scrutinized (135, 136), which represents a new application area where improved MD and TD MS methods will be valuable.

## Conflict of interest

The authors declare the following competing financial interest(s): N.A.K is an employee of Spectrotech SAS and A. N. K., K. O. N., and Y. O. T. are employees of Spectroswiss Sarl. Both companies develop and commercialize FTMS data acquisition, processing, and analysis tools.
